# Effects of milk product intake on thigh muscle strength and *NFKB* gene methylation during home-based interval walking training in older women: A randomized, controlled pilot study

**DOI:** 10.1371/journal.pone.0176757

**Published:** 2017-05-17

**Authors:** Shizue Masuki, Kensei Nishida, Shigenari Hashimoto, Mayuko Morikawa, Satoshi Takasugi, Masashi Nagata, Shun'ichiro Taniguchi, Kazuhito Rokutan, Hiroshi Nose

**Affiliations:** 1 Department of Sports Medical Sciences, Shinshu University Graduate School of Medicine, Matsumoto, Japan; 2 Institute for Biomedical Sciences, Shinshu University, Matsumoto, Japan; 3 Department of Pathophysiology, Institute of Biomedical Sciences, Tokushima University Graduate School, Tokushima, Japan; 4 Advanced Preventive Medical Center, Shinshu University Hospital, Matsumoto, Japan; 5 Jukunen Taiikudaigaku Research Center, Matsumoto, Japan; 6 Food Science Research Laboratories, Meiji Co. Ltd., Odawara, Japan; 7 Department of Molecular Oncology, Shinshu University Graduate School of Medicine, Matsumoto, Japan; Victoria University, AUSTRALIA

## Abstract

**Background:**

Muscle atrophy with aging is closely associated with chronic systemic inflammation and lifestyle-related diseases. In the present study, we assessed whether post-exercise milk product intake during 5-month interval walking training (IWT) enhanced the increase in thigh muscle strength and ameliorated susceptibility to inflammation in older women.

**Methods:**

Subjects [n = 37, 66±5 (standard deviation) yrs] who had been performing IWT for >6 months participated in this study. They were randomly divided into the following 3 groups: IWT alone (CNT, n = 12), IWT + low-dose post-exercise milk product intake (LD, n = 12; 4 g protein and 3 g carbohydrate) or IWT + a 3-times higher dose of milk product intake than the LD group (HD, n = 13). They were instructed to repeat ≥5 sets of fast and slow walking for 3 min each at ≥70% and 40% peak aerobic capacity for walking, respectively, per day for ≥4 days/week.

**Results:**

After IWT, thigh muscle strength increased in the HD group (8±2%) more than in the CNT group (-2±3%, *P* = 0.022), despite similar IWT achievements between the groups (*P*>0.15). Pyrosequencing analysis using whole blood showed that methylation of *NFKB1* and *NFKB2*, master genes of inflammation, was enhanced in the HD group (29±7% and 44±11%, respectively) more than in the CNT group (-20±6% and -10±6%, respectively; *P*<0.001). Moreover, the genome-wide DNA methylation analysis showed that several inflammation-related genes were hyper-methylated in the HD group compared with that in the CNT group, suggesting greater pro-inflammatory cytokine gene suppression in the HD group.

**Conclusion:**

HD milk product intake after exercise produced a greater percent increase in thigh muscle strength and *NFKB1* and *NFKB2* gene methylation during IWT in physically active older women.

**Trial registration:**

UMIN-CTR No. UMIN000024544 and No. UMIN000024912

## Introduction

It has been suggested that physical fitness deterioration mainly due to muscle atrophy with advanced aging (sarcopenia) is closely associated with chronic systemic inflammation and age- and lifestyle-related diseases (LSDs) [1–5]. To prevent such deterioration, exercise training and/or nutritional supplementation has been recommended for middle-aged and older people [6, 7]; however, no broadly available regimens have been established.

To solve this problem, we have developed an exercise training program that is broadly applicable for middle-aged and older people and has only minimal personnel and financial requirements. The program comprises interval walking training (IWT) and involves the use of an information technology network system that tracks exercise intensity and energy expenditure during training [8–10]. Using this system, we reported that ~5 months of IWT increased thigh muscle strength and peak aerobic capacity for walking ($\dot{V}O_{2\mathrm{peak}}$) by ~10% in middle-aged and older people [8, 11], which was accompanied by improved LSD symptoms by ~20% [8, 11] and increased DNA methylation (inactivation) of the *NFKB2* gene, one of the master pro-inflammatory response genes [12]. These findings suggest that IWT ameliorates susceptibility to inflammation with increasing physical fitness in middle-aged and older people.

On the other hand, nutritional supplementation studies suggest that daily milk product intake suppresses inflammatory responses and prevents LSDs [13, 14]. However, during the above interventions, because exercise intensity and energy expenditure were not monitored, it remains unknown how physical activity is involved in the effects of milk product intake. Notably, we recently found that a mixture of milk-protein and carbohydrate supplementation during 5 months of IWT increased thigh muscle strength more than IWT alone in middle-aged and older women [15]. In addition, muscle atrophy with aging is associated with chronic systemic inflammation [2–5], and increases in muscle strength elicited by regular exercise are accompanied by decreased NF-κB activity in older men [16]. If this is the case, milk-protein and carbohydrate intake during exercise training would have synergistic effects on suppression of chronic systemic inflammation as well as protect against age-associated declines in muscle strength.

Accordingly, in the present study, we examined the hypothesis that cheese and yogurt intake during IWT would enhance methylation of *NFKB* genes and other related pro-inflammatory cytokine genes in conjunction with an increase in thigh muscle strength in middle-aged and older people. The reason for the use of cheese and yogurt as a supplement is that they are less expensive and more readily available to the general population than the previously used supplements [15, 17, 18]. If we obtain results supporting this hypothesis, the regimen could be broadly accepted by middle-aged and older people aiming to prevent LSDs associated with age-related declines in physical fitness.

## Methods

### Trial design

This study was carried out in a randomized controlled design. The protocols for this study and supporting CONSORT checklist are available as supporting information; see S1 Checklist and S1–S4 Protocols.

### Ethics statement

This study was approved by the Review Board on Human Experiments, Shinshu University School of Medicine and conformed to the standards set by the Declaration of Helsinki. We obtained written informed consent from all participants involved in this study.

### Trial registration

The trial was registered in UMIN after participant recruitment for this study. The reason for the delay in registering was that we were unaware of the importance of registration preceding recruitment. However, we recruited the first participant for this study after approval by the Review Board on Human Experiments, Shinshu University School of Medicine (approval date: February 7, 2012). Additionally, the authors confirm that all ongoing and related trials for this intervention are registered.

### Subjects

**Fig 1:**
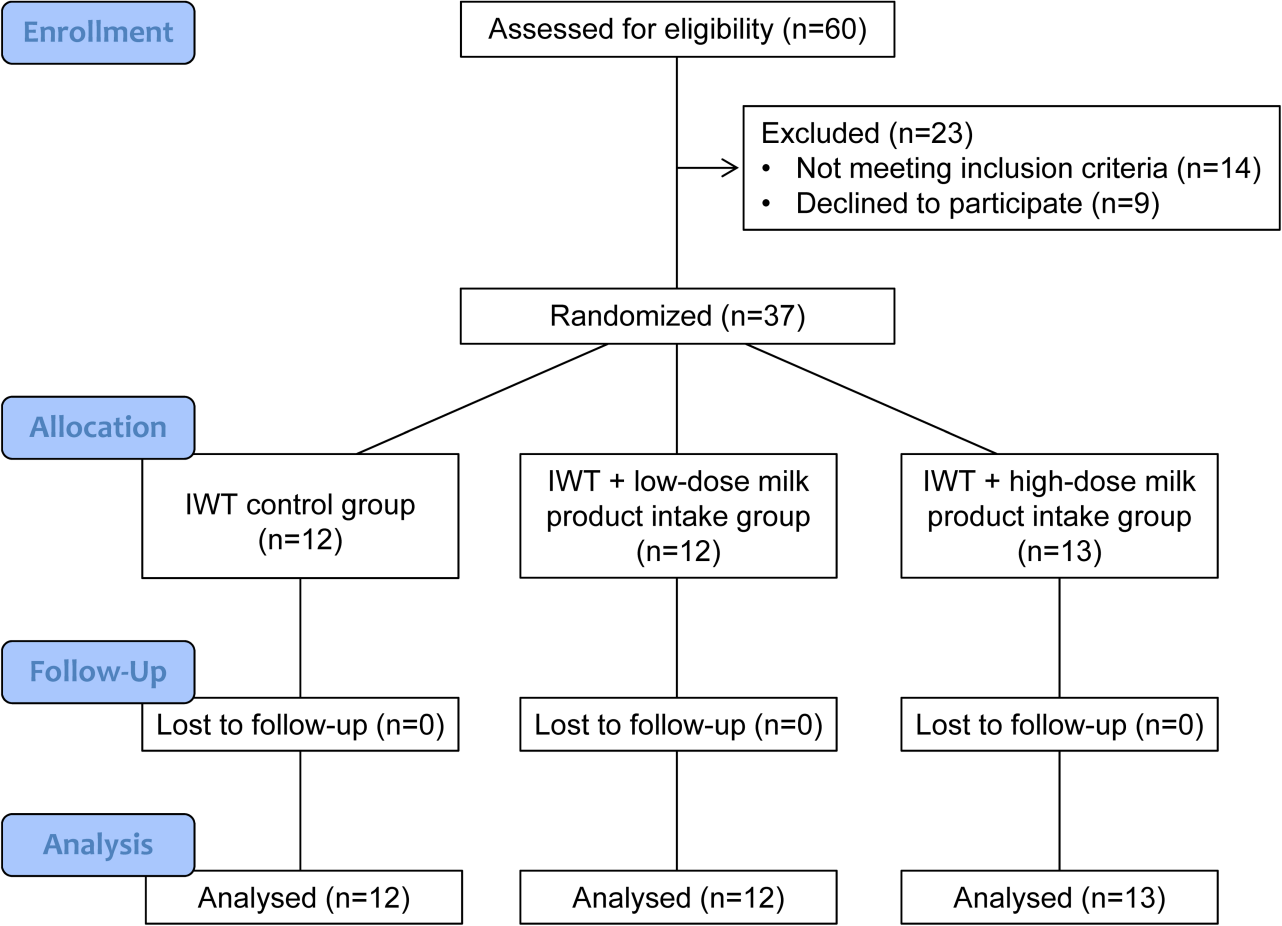
CONSORT flow diagram. IWT, interval walking training.

After receiving a full explanation of the experimental protocol, 37 healthy female volunteers (55–75 years of age) provided written informed consent before participating in this study (Fig 1). The subjects were recruited from among the 527 participants in the “Jukunen Taiikudaigaku Project”, a health promotion program for middle-aged and older people in Matsumoto City, Japan. The recruitment was performed from February 8 to March 31, 2012. We recruited female subjects to minimize any confounding effects of gender. We also recruited subjects who had performed the IWT program for more than 6 months prior to this study because they were familiar with the exercise testing procedures used in the present study and because their improvements in physical fitness and LSD risk factors had likely reached a steady state; therefore, we surmised that we could detect any effects elicited by post-exercise milk product intake in addition to those of IWT alone. Each subject provided a complete medical history and underwent a physical examination. All subjects were nonsmokers, had no overt history of hepatic, thyroid, renal, metabolic, cardiovascular, or pulmonary disease, had no orthopedic limitations that could affect exercise testing or training and had no gastrointestinal symptoms related to milk product intake. All subjects participated in both clinical and genetic assessments. Ultimately, 14 females were excluded because of knee pain (n = 2), lactose intolerance (n = 1), or failure to participate in genetic assessments (n = 11).

### Randomization

Subjects were randomly assigned to one of 3 groups by an independent investigator (H.G.) using permuted-block randomization (block size: 6) with an allocation ratio of 1:1:1. The investigator was not involved in participant recruitment or any assessments. The random allocation sequence was generated using a computer.

### Protocol

The experiments were conducted from April 6 to September 20, 2012. Subjects were instructed to refrain from vigorous exercise on the day before measurements were taken. They arrived at a gym at 09:00 on their assigned day in April following an overnight fast. We measured their physical characteristics and sampled their blood for determination of blood lipid and glucose levels, as well as DNA methylation levels. After subjects had a light breakfast and rested for 1 hour, we measured thigh muscle strength and $\dot{V}O_{2\mathrm{peak}}$ for walking. As stated above, the subjects were randomly divided into the following 3 groups: IWT alone (CNT, n = 12), IWT + low-dose post-exercise milk product intake (LD, n = 12) or IWT + high-dose post-exercise milk product intake (HD, n = 13) (see below for details of milk product intake) to evaluate the dose-dependent effects. There were no significant differences in physical characteristics or fitness among the groups (Table 1).

**Table 1:** Physical characteristics and fitness at baseline and changes after training.

		Before			%Change	
	CNT (n = 12)	LD (n = 12)	HD (n = 13)	CNT (n = 12)	LD (n = 12)	HD (n = 13)
Age, yr	66±4	66±5	67±6	NA	NA	NA
Height, cm	153±4	155±4	154±5	NA	NA	NA
Body weight, kg	54.9±2.2	57.7±2.9	55.8±2.1	-0.9±0.7*	-1.7±0.7*	-0.3±0.6
BMI, kg/m^2^	23.5±0.8	23.9±0.9	23.4±0.7	-0.9±0.7*	-1.7±0.7*	-0.3±0.6
F_EXT_, N	373±23	396±30	369±34	-6.2±4.3	3.5±4.3	4.1±4.1
F_FLX_, N	188±7	203±15	180±10	2.9±5.5	0.2±2.9	11.8±5.1
$\dot{V}O_{2\mathrm{peak}}$, ml/min	1378±74	1625±120	1337±56	13.1±2.5***	7.1±2.6***	9.2±2.4***
HR_peak_, beats/min	141±6	145±5	140±5	2.9±2.2	2.4±2.1	1.9±2.0

Values are the mean ± standard deviation for age and height and the mean ± standard error (SE) for other variables. CNT, interval walking training control group; LD and HD, interval walking training + low- and high-dose milk product intake groups, respectively; NA, not applicable; F_EXT_, isometric knee extension force; F_FLX_, isometric knee flexion force; $\dot{V}O_{2\mathrm{peak}}$, peak aerobic capacity for walking; HR_peak_, peak heart rate at $\dot{V}O_{2\mathrm{peak}}$. % Change was calculated as (after-before)/before x 100 and then adjusted for pretraining values by ANCOVA. Significant differences from pretraining values, * *P*<0.05 and *** *P*<0.001.

Subjects in all groups performed IWT for 5 months. This period was chosen based on previous studies suggesting that ~5 months of IWT increased $\dot{V}O_{2\mathrm{peak}}$ and thigh muscle strength [8], improved the symptoms of LSDs [11], and altered DNA methylation of pro-inflammatory genes [12]. In addition, Okazaki et al. [15] suggested that a mixture of milk-protein and carbohydrate intake during 5 months of IWT enhances the increase in thigh muscle mass and strength in middle-aged and older people. During the 5-month training period, the average atmospheric temperature ranged from 16 to 26°C, and the average relative humidity ranged from 62 to 73%. All measurements performed before training were repeated after training using the same procedures. All subjects completed 5 months of training without any harmful events and returned for a post-training assessment.

### IWT regimen

As reported previously [8], all subjects were instructed to repeat ≥5 sets of 3 min of low-intensity walking at ~40% of their pre-training $\dot{V}O_{2\mathrm{peak}}$ for walking (see below for details), followed by 3 min of high-intensity walking at ≥70% of their pre-training $\dot{V}O_{2\mathrm{peak}}/\mathrm{day}$ for ≥4 days/week. Energy expenditure during daily walking at their favorite time and place was monitored with a portable calorimeter (Jukudai Mate; Kissei Comtec, Matsumoto, Japan) on the right or left side of the waist at the midclavicular line. A beeping signal alerted the participants when a change in intensity was scheduled, and another signal alerted them when their walking intensity reached 70% $\dot{V}O_{2\mathrm{peak}}$. Additionally, the device informed them of their total fast walking time per IWT at the end of each training session.

The calorimeter was equipped with a tri-axial accelerometer and a barometer to monitor the vector magnitude and changes in altitude, respectively. Using these variables, Yamazaki et al. [9] developed the logic to estimate energy expenditure precisely during walking even when subjects walk on inclines. Regarding the precision to estimate $\dot{V}O_{2}$ (ml/kg/min) from this logic, they suggested that the estimated $\dot{V}O_{2}(y)$ was almost identical to the measured $\dot{V}O_{2}(x)$, which was pooled from 11 subjects who walked on inclines in the field with varied slopes (*y* = 0.969*x*, r = 0.879, *P*<0.001); the mean difference was -0.20 ml/kg/min, and the 95% prediction limits were ±6.95 ml/kg/min over the range of 2.0–33.0 ml/kg/min in the Bland-Altman analysis [9]. Thus, the exercise intensity during IWT measured with the device was reliable enough to detect any significant differences in the training achievement among the groups in the present study.

Every 2 weeks, the subjects visited a local community office near their homes to upload their walking records from the calorimeter to a central server at the administrative center via the Internet for automatic analysis and reporting. The trainers used these reports to track daily walking intensity, energy expenditure, and other parameters, which are shown in Table 2, and to instruct the participants on how best to achieve their target levels. The target intensity for walking was not re-adjusted during the 5-month training period.

**Table 2:** Training achievements over 5 months.

	CNT (n = 12)	LD (n = 12)	HD (n = 13)
Walking days per week	3.9±0.3	4.4±0.2	4.5±0.2
Fast walking			
Time, min/day	19±1	21±1	22±2
§Energy expenditure, mlO_2_/walking day	19,681±1,509	24,561±2,215	20,860±1,731
§Intensity, mlO_2_/min	1,014±53	1,206±106	964±52
Slow walking			
Time, min/day	34±5	23±2	26±3
§Energy expenditure, mlO_2_/walking day	17,268±2,260	14,308±1,016	14,518±1,752
§Intensity, mlO_2_/min	544±35	631±36	568±31

Values are the mean ± SE.

§ Resting oxygen consumption is not included.

### Milk product intake after IWT

Subjects in the LD and HD groups were instructed to ingest milk products within 30 min after completing >15 min of fast walking for IWT with no intermission. The nutritional components of the milk products are shown in Table 3. Subjects in the LD group ingested either single alternating units of cheese or yogurt, whereas those in the HD group ingested 1 unit of cheese + 2 units of yogurt. Subjects in the CNT group were not provided with milk products.

**Table 3:** Nutritional components of the milk products.

	Post-exercise milk product intake	
	LD§ (1 unit of cheese or yogurt/dose)	HD (1 unit of cheese + 2 units of yogurt/dose)
Energy, kcal	60	171
Protein, g	4.1	12.3
Carbohydrate, g	2.5	9.4
Fat, g	3.7	9.4

The amounts representing 1 unit of cheese and yogurt were 18.4 and 80.0 g, respectively. Subjects in the LD and HD groups ingested the milk product(s) within 30 min after completing >15 min of fast walking for interval walking training with no intermission. On average, subjects in the LD and HD groups consumed the milk products 80±2 and 77±2 times during the 5-month training period, respectively.

§ Subjects in the LD group ingested single alternating units of cheese or yogurt; therefore, the values in the LD group represent the average of the 2 products/unit.

We chose this dose of milk products based on a previous study that showed that milk protein (8 g) and carbohydrate (33 g) supplementation immediately after exercise during IWT promoted increases in skeletal muscle mass and strength in middle-aged and older women [15]. Additionally, milk protein (10–12 g) and carbohydrate (15–35 g) supplementation immediately after exercise was reported to enhance plasma volume expansion and, thereby, facilitate thermoregulatory adaptation in older men [19, 20]. Thus, the timely supplementation of adequate amounts of milk protein and carbohydrate might improve age-associated deterioration in homeostatic regulation by providing tissues with required substrates after exercise. Therefore, we speculated that if we provide milk product(s) that contain similar amounts of protein and carbohydrate to previous studies immediately after exercise, it would make it possible to assess our hypothesis, even though its impact on total protein intake per day might not be massive.

Subjects in all groups were instructed to refrain from eating and drinking any foods and fluids other than tap water or the assigned milk product(s) during and for at least 60 min after each IWT session. To examine adherence to the intervention, all subjects were required to keep daily logs describing whether they completed IWT; in addition, the subjects in the LD and HD groups reported whether they ingested the above milk product(s) as instructed. Using this information, adherence to the post-exercise milk product intake regimen was determined by dividing the number of days in which the subjects ingested milk product(s) as instructed by the number of days in which the subjects accomplished >15 min of fast walking for IWT with no intermission. When the subjects walked more than 4 days/week, these walking days were not included in the analysis because milk products were not provided in these days.

### Dietary intake

All subjects were instructed to maintain their dietary habits during the study period. In addition, subjects were instructed to report the foods that they consumed for 7 consecutive days during the training period in May, July, and September by answering a questionnaire that was prepared by a dietician (FFQg Ver 3.5; Kenpakusya, Tokyo, Japan). The results are shown in Table 4.

**Table 4:** Dietary intake per day during the training period.

	CNT (n = 12)	LD (n = 12)	HD (n = 13)
Total energy, kcal	1,603±54	1,691±60 (1,725±60)	1,606±64 (1,700±63)
Protein, g	57.3±2.9	62.3±3.1 (64.6±3.1)	58.1±2.8 (64.9±2.8)
Protein per body weight, g/kg	1.1±0.1	1.1±0.1 (1.2±0.1)	1.1±0.1 (1.2±0.1)
Carbohydrate, g	229±7	228±7 (229±7)	222±8 (227±8)
Fat, g	47.1±3.3	53.1±2.9 (55.3±2.8)	47.8±2.6 (53.0±2.6)
Energy for milks§ among total energy, kcal	123±21	142±19 (176±19)	108±12 (203±13††)

Values are the mean ± SE.

§ Milks indicates milk and milk products. The first set of values do not include milk product intake after training, whereas the values in parentheses include milk product intake after training.

†† Significant difference from the corresponding value in the CNT group, *P*<0.01.

### Measurements

#### Thigh muscle strength

Isometric knee extension (F_EXT_) and flexion (F_FLX_) forces were measured twice in the dominant leg with an isometric force meter (GT330, OG Giken, Okayama, Japan) by the staff (who were blinded to subject groupings), and the higher value was used for analysis.

#### $\dot{V}O_{2\mathrm{peak}}$

On the same day as the muscle strength measurements, $\dot{V}O_{2\mathrm{peak}}$ was determined by measuring energy expenditure with the calorimeter (Jukudai Mate; Kissei Comtec, Matsumoto, Japan) during graded-intensity walking on a flat floor at subjectively slow, moderate, and the fastest speeds for 3 min each, as reported previously [9]. Regarding the precision of the measurement, we confirmed that the $\dot{V}O_{2\mathrm{peak}}$ (ml/min) obtained with the calorimeter during the graded walking exercise was highly correlated with the $\dot{V}O_{2\mathrm{peak}}$ obtained by graded cycling exercise with respiratory gas analysis (R^2^ = 0.83, *P*<0.0001). Furthermore, the regression coefficient was close to a unit in middle-aged and older men and women (n = 278) and within ±15 ml/min of the 95% confidence limit over the range of $\dot{V}O_{2\mathrm{peak}}$ variation [8]. These results suggest that the $\dot{V}O_{2\mathrm{peak}}$ for walking, as determined by the graded walking test, was reliable enough to detect any significant differences in the increase in peak aerobic capacity after training between the groups in the present study.

#### Blood samples

Blood samples were collected from the antecubital vein to measure blood lipid and glucose levels and to extract DNA before and after training in all subjects. Serum cholesterol, triglyceride, and plasma glucose concentrations were determined using standard enzymatic methods. Genomic DNA was extracted using the QIAamp DNA blood Mini kit (Qiagen, Hilden, Germany), according to the manufacturer’s instructions.

### Analyses

#### Thigh muscle strength analysis

In the present study, we averaged the percent increase in F_EXT_ and F_FLX_ after training to determine the overall improvement in thigh muscle strength in each subject [21]. We used this approach because we found an increase in either F_EXT_ or F_FLX_ in 70% of the subjects (see Results for details). This may be due to the varied landform, which was either flat ground or inclines, where the subjects performed the IWT [22]. Similar to our approach, in a previous bicycle training study [21], the power values averaged for isokinetic knee extension and flexion were used as leg power on a dynamometer. The authors reported that the power on the dynamometer before and after training (when pooled) was significantly correlated with the maximal power output that could be maintained on the bicycle ergometer. Additionally, other studies have also used the average of the knee extension and flexion [23] or the combination of knee extension and flexion scores [24]. Together, the above observations suggest that combined F_EXT_ and F_FLX_ are likely to provide some insight into overall leg strength.

#### DNA methylation by pyrosequencing

Because *NFKB* genes play a key role in inflammation [25] and are suggested to be inactivated through DNA methylation after ~6 months of IWT [12], we examined the effects of milk product intake during IWT on DNA methylation of the *NFKB1* and *NFKB2* genes.

**Fig 2:**
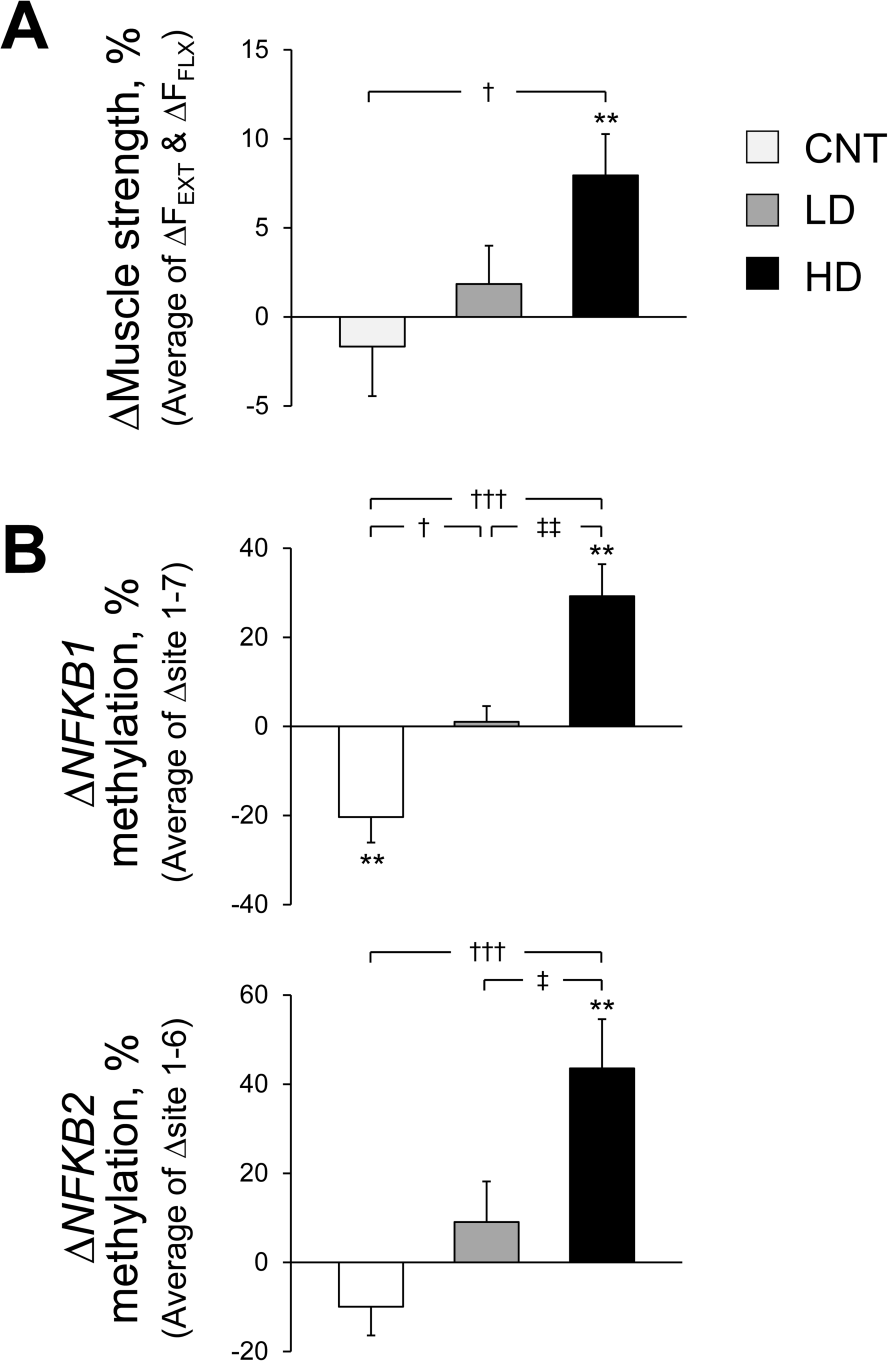
**Percent changes after training in muscle strength (A) and methylation of the *NFKB1* and *NFKB2* promoter regions assessed by pyrosequencing (B)**. The data were adjusted for pretraining values by ANCOVA. The mean and SE bars are presented for 12, 12, and 13 subjects in the IWT control (CNT), IWT + low-dose (LD) and IWT + high-dose milk product intake (HD) groups, respectively. **Significant differences from pretraining values, *P*<0.01. Significant differences from the CNT group, †*P*<0.05 and †††*P*<0.001. Significant differences from the LD group, ‡*P*<0.05 and ‡‡*P*<0.01. **A**: Average percent changes in isometric knee extension (ΔF_EXT_) and flexion (ΔF_FLX_) forces are presented. **B**: Average percent changes in CpG sites 1–7 for *NFKB1* (*upper*) and average percent changes in CpG sites 1–6 for *NFKB2* (*lower*) are presented.

We analyzed samples from all subjects in the CNT (n = 12), LD (n = 12), and HD (n = 13) groups before and after training by pyrosequencing (PyroMark Q24ID; Qiagen, Hilden, Germany). PCR and sequencing primers were designed using PyroMark Assay Design 2.0 software (Qiagen), and all procedures were performed according to the recommended protocols. The promoter regions of *NFKB1* and *NFKB2* (-443 to -101 and -1281 to -1018 upstream of the transcription start site, respectively) were amplified by PCR. The primers are shown in Table 5. DNA methylation predominantly occurs on cytosines at sites of CpG dinucleotides in mammals; therefore, the target regions of *NFKB1* and *NFKB2* were 5’- CGCAGGGGCCGCGGCGTCCAGGCCGCCTAACGCG -3’ and 5’- AAAGGGCGCGAGGCGTGACGCACGGAAACGTCA -3’, respectively (-240 to -207 and -1238 to -1206 upstream of the transcription start site, respectively). Briefly, bisulfite conversion of 500 ng of genomic DNA was performed with an EpiTect kit (Qiagen). Bisulfite-converted DNA was amplified by PCR with a forward primer for *NFKB1* and a reverse primer for *NFKB2* biotinylated at its 5’ end using a PyroMark PCR Master Mix kit (Qiagen). Biotinylated PCR products were immobilized onto streptavidin-coated beads (GE Healthcare, Uppsala, Sweden), and the DNA strands were separated using denaturation buffer. After washing and neutralization at a PyroMark Q24 Vacuum Workstation, the sequencing primer was annealed to the immobilized strand. DNA methylation was analyzed via highly quantitative bisulfite pyrosequencing with a PyroMark Q24 system (Qiagen). Data were analyzed using PyroMark Q24 software (Qiagen), and the results are presented in Fig 2.

**Table 5:** Primers used for the pyrosequencing assay.

Gene	Amplification site	Forward primer (5’ to 3’)	Reverse primer (5’ to 3’)	Sequencing primer (5’ to 3’)
*NFKB1*	-443 to -101	Biotin-GGGGGAAGTTAGTATTTTTAGGGG	TCCATACCCACCCTCCAACTACT	-258-ACTAAAAAACCAAAACCC
*NFKB2*	-1281 to -1018	GGGTTGGTTGAGTTAGTTTAGAGTTAAAT	Biotin-CCTCCTCCCTCTTTTCTCTTATCC	-1262-AGAGTTAAATTTTTAGTTAATGAA

#### Genome-wide DNA methylation by the Infinium 450K methylation assay

In the present study, we found that milk product intake during IWT significantly increased methylation of the *NFKB1* and *NFKB2* genes, which are master genes of inflammation [25] (see Results for details); therefore, we also examined how milk product intake affected global methylation profiles including pro-inflammatory-related genes via the genome-wide DNA methylation analysis. The samples used for this assay were obtained from the CNT and HD groups but not from the LD group to simplify the comparisons. Samples included all 12 subjects in the CNT group and 12 randomly selected subjects of the 13 in the HD group before and after training in the same subjects. The reason for the selection of 12 subjects in the HD group was that this assay was limited to a set of 12 samples. The selection was performed by an independent investigator (H.G.) who was not involved in any assessments.

**Fig 3:**
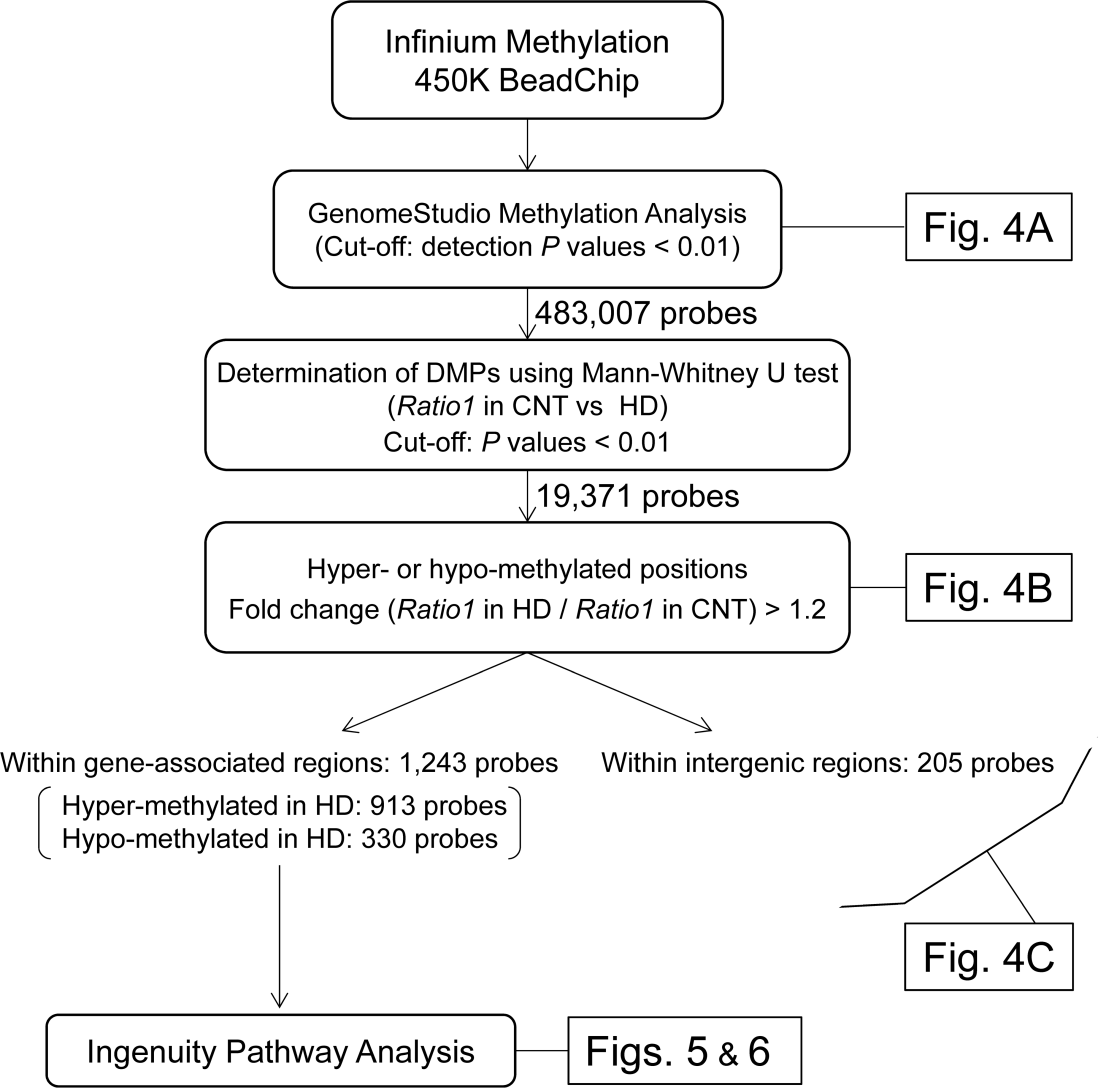
Flow chart diagram showing the genome-wide DNA methylation analysis. DMPs, differentially methylated CpG positions; *Ratio1*, the ratio of the β-value after training to that before training.

The genome-wide DNA methylation analysis process is shown in Fig 3. Briefly, we determined more hyper- or hypo-methylated positions after training in the HD group than those in the CNT group using the following procedure. First, bisulfite conversion of 500 ng of genomic DNA was performed with the EZ DNA methylation-gold kit (Zymo Research, Irvine, CA), and then, the bisulfite-converted DNA was subjected to the Infinium HumanMethylation450 BeadChip array (Illumina Inc., San Diego, CA) to determine the methylation levels of the CpG sites. This array provides highly accurate and highly reproducible results via an established technical scheme [26–28], in which technical validation studies showed a strong correlation coefficient of 0.99 between array replicates [26, 29]. Using this array, we confirmed that 485,764 CpG dinucleotides covered 96% of the CpG-rich regions (CpG islands) from the UCSC database and, moreover, covered CpG island shores (0–2 kb from CpG islands) and CpG island shelves (2–4 kb from CpG islands) using quantitative high-throughput DNA methylation analysis.

Second, the DNA methylation levels of the CpG sites on the array were evaluated by the β-values, which represented the percentage of methylation ranging from 0 (completely unmethylated) to 1 (fully methylated). This was conducted using GenomeStudio software (Illumina Inc.) after color balance adjustment and background corrections were performed on every set of 12 samples in the same chip for intra-chip normalization.

**Fig 4:**
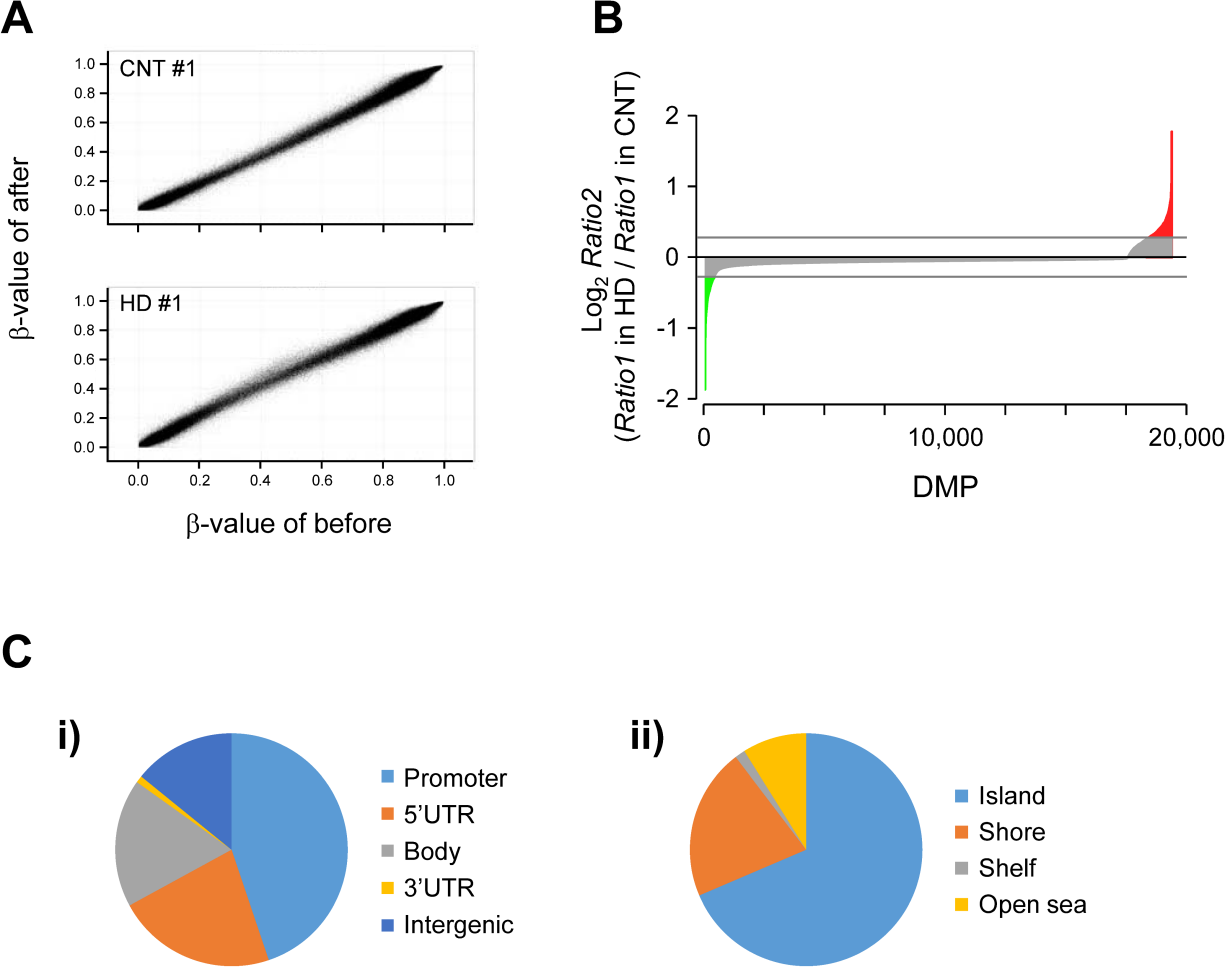
The hyper- or hypo-methylated positions induced by milk product intake during IWT in whole blood cells. **A**: DNA methylation levels of CpG sites identified by the Infinium 450K methylation assay. Typical examples of normalized β-values before vs after training in a CNT subject and an HD subject, which were used to determine the ratio (*Ratio1*) of the β-value after training to that before training in each CpG site. **B:** *Ratio2* on a base-2 logarithmic scale (*y*) plotted against 19,371 differentially methylated CpG positions (DMPs) after training in the HD group (n = 12) compared with the CNT group (n = 12) (*x*). *Ratio2* indicates the ratio of the median value of *Ratio1* for the HD group to that for the CNT group at each DMP. The number of DMPs on the *x*-axis is an arbitrary unit, which was ranked by the *Ratio2* values. The upper and lower areas of the gray lines in the figure indicate the *Ratio1* values of the HD that group are >1.2-fold higher (indicated by red) and lower (indicated by green) than that of the CNT group, respectively. **C:** Pie chart displaying the genomic location of the 1,448 positions in which more hyper- or hypo-methylation occurred in the HD group than in the CNT group by >1.2-fold in relation to genes (i) and CpG (ii) context.

**Fig 5:**
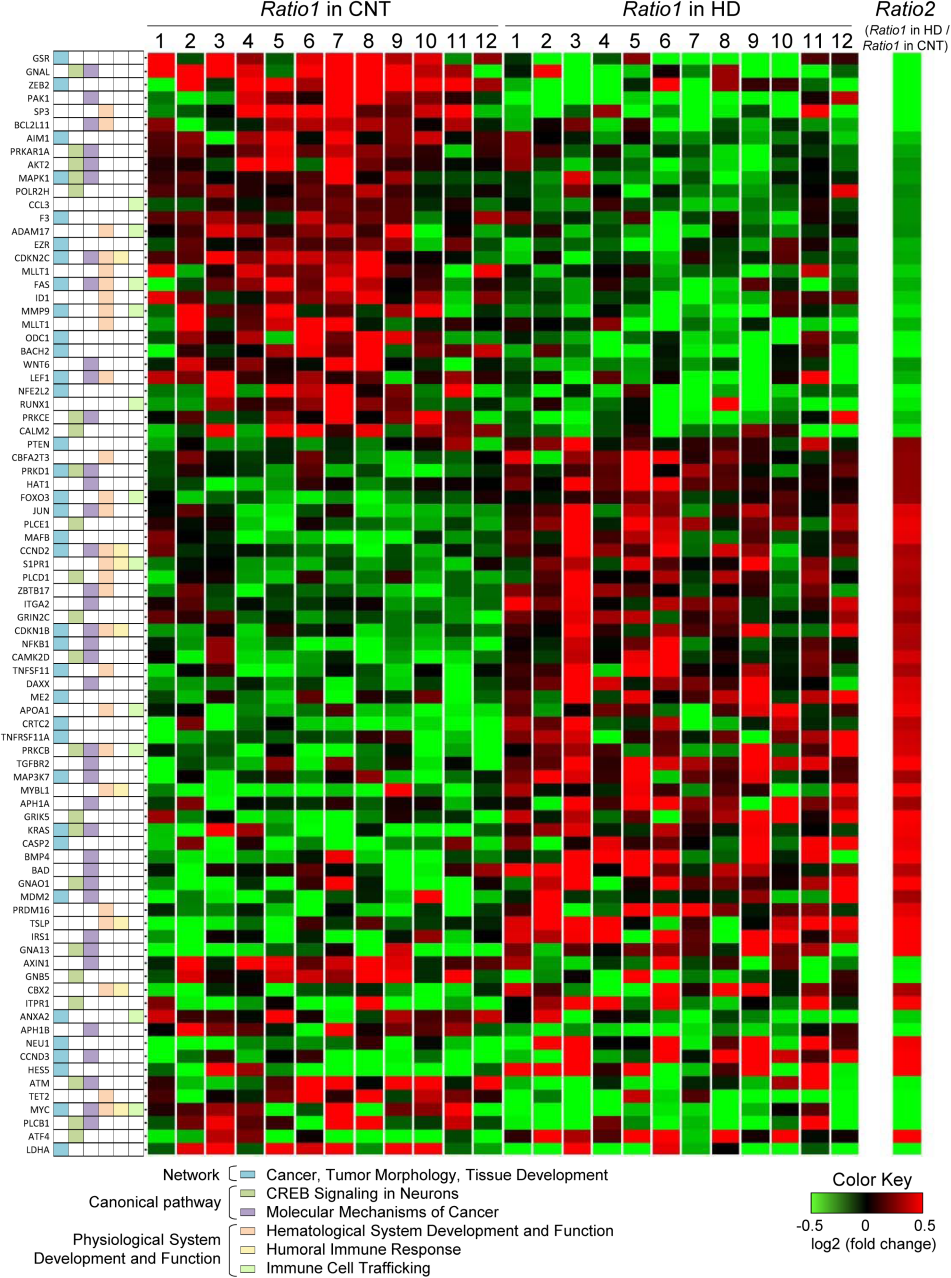
Hierarchical clustering of the hyper- or hypo-methylated positions induced by milk product intake during IWT. Among the 1,448 positions in which more hyper- or hypo-methylation occurred in the HD group than in the CNT group by >1.2-fold, those within gene-associated regions (1,243 positions) were subjected to an ingenuity pathway analysis (IPA). Hierarchical clustering analysis was then performed on the dataset obtained from the results of the IPA, which included 83 genes in the top network, canonical pathways, or physiological functions.

**Fig 6:**
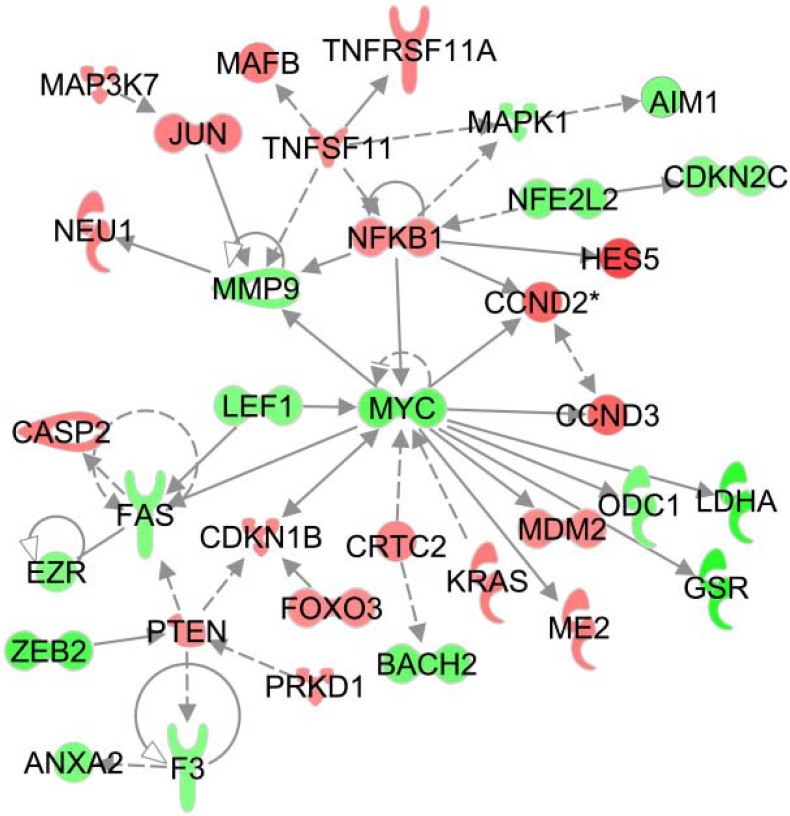
IPA-derived top-scoring network: a cancer-, tumor morphology-, and tissue development-related network (score = 42). Colored genes are hyper- (red) or hypo-methylated (green). A solid line indicates a direct interaction, and a dashed line indicates an indirect relationship between nodes. The shape of the node indicates its function. More information is available at the Ingenuity Inc. website (http://www.ingenuity.com).

Finally, for the quality guarantee, we selected CpG positions in which β*-*values were detected at the level of *P* <0.01 [30] and determined the data set of 483,007 CpG sites on autosomal and X chromosomes, which we used as qualified CpG sites in the following statistical analyses (Figs 4–6). We deposited our genome-wide DNA methylation data into the Gene Expression Omnibus (GEO) of the National Center for Biotechnology Information under the accession number GSE76503.

### Statistics

Analyses were performed using IBM SPSS Statistics 22 (Armonk, NY). The Shapiro-Wilk test was used to check distributions of the data for normality prior to applying the analysis of variance (ANOVA) and covariance (ANCOVA). A one-way ANOVA was used to examine any significant differences in physical characteristics, thigh muscle strength, $\dot{V}O_{2\mathrm{peak}}$, and *NFKB1* and *NFKB2* methylation before training and their changes after training among the groups (Tables 1 and 6). This model was also used to examine any significant differences in training achievements and dietary intake during the training period among the groups (Tables 2 and 4). A two-way ANOVA for repeated measures was used to examine any significant effects of training on the variables in each group (Tables 1 and 6, Fig 2). Moreover, we examined any significant differences in their changes after training among the groups by ANCOVA, with the pre-training values for each subject included as covariates (Tables 1 and 6, Fig 2). The Tukey-Kramer test was used as a subsequent post hoc test to perform any pairwise comparisons among the groups. Additionally, we present the minimum sample sizes to detect group differences in their percent changes after training for key variables in the present study—thigh muscle strength and methylation of the *NFKB1* and *NFKB2*.

**Table 6:** Methylation of the *NFKB1* and *NFKB2* promoter regions at baseline and changes after training assessed by pyrosequencing.

		Before			%Change	
	CNT (n = 12)	LD (n = 12)	HD (n = 13)	CNT (n = 12)	LD (n = 12)	HD (n = 13)
*NFKB1* methylation, %cytosine methylated						
CpG site 1	5.7±0.3	6.0±0.3	5.5±0.3	-7.3±7.1	-10.9±7.3	11.0±7.2‡
2	7.3±0.8	7.9±0.9	7.8±1.0	-32.4±9.8**	12.7±9.1*††	23.6±7.4**†††
3	12.8±0.4	12.5±0.4	11.6±0.6	-16.8±4.8**	8.4±4.3*†††	23.9±4.1***†††‡‡
4	4.6±0.3	5.1±0.3	4.8±0.4	-15.3±8.4	10.6±8.0	27.3±7.2**†††
5	10.9±0.5	11.9±0.7	9.9±0.5	-23.3±9.8*	-3.9±7.9	18.4±7.8*††‡
6	10.4±0.6	11.6±0.6	9.8±0.6	-19.2±11.0	-0.4±10.1	21.5±9.6†
7	6.3±0.6	6.3±0.3	5.7±0.2	-28.1±21.5***	-9.3±21.1	78.9±23.1*††‡
*NFKB2* methylation, %cytosine methylated						
CpG site 1	25.7±1.7	25.3±1.4	27.2±2.5	-10.3±9.4	8.5±9.4	42.8±9.0**†††‡
2	25.7±1.6	25.3±1.5	26.6±2.4	-9.6±9.5	9.1±9.5	43.6±9.2**†††‡
3	27.7±1.7	27.8±1.6	28.5±2.6	-9.5±9.4	9.3±9.4	40.1±9.1**†††‡
4	24.8±1.5	24.8±1.5	26.5±2.4	-9.6±9.3	10.6±9.3	43.8±9.0**†††‡
5	21.8±1.3	20.8±1.2	23.4±2.2	-11.1±9.8	7.5±9.9	48.0±9.5**†††‡‡
6	25.8±1.6	25.8±1.5	27.3±2.5	-9.5±9.6	9.5±9.6	43.0±9.3**†††‡

Values are the mean ± SE. The target regions of *NFKB1* and *NFKB2* were located -240 to -207 and -1236 to -1203 upstream of the transcription start site, respectively. % Change was calculated as (after-before)/before x 100 and then adjusted for pretraining values by ANCOVA. Significant differences from pretraining values, * *P*<0.05, ** *P*<0.01 and *** *P*<0.001.

Significant differences from the corresponding values in the CNT group, † *P*<0.05, †† *P*<0.01, and ††† *P*<0.001.

Significant differences from the corresponding values in the LD group, ‡ *P*<0.05 and ‡‡ *P*<0.01.

For the genome-wide DNA methylation analysis, the Mann-Whitney U test was used to identify differentially methylated CpG positions (DMPs) after training in the HD group compared with that of the CNT group (Fig 4). For this analysis, *P-*values <0.01 were considered significant.

χ^2^ analysis was used to examine any significant differences in the pattern of changes in muscle strength after training: either F_EXT_ or F_FLX_ increased/decreased or both increased/decreased. *P*-values <0.05 were considered significant, except for those pertaining to the microarray data (Figs 4–6) as stated above. Values are expressed as the mean ± standard error (SE) unless otherwise indicated.

## Results

### Adherence to the exercise training program

As shown in Table 2, there were no significant differences in training achievement among the CNT, LD, and HD groups. All groups achieved or exceeded the target of 4 training sessions per week, with no differences among the groups. Moreover, for each group, the average fast walking time was higher than the target prescribed before training (15 min/walking day). Thus, our subjects had high adherence to the 5-month IWT program.

### Adherence to post-exercise milk product intake

Before starting training, we instructed the subjects in the LD and HD groups to ingest milk products only after they had completed >15 min of fast walking for IWT with no intermission. Adherence to the post-exercise milk product intake regimen was 99.8±0.1% and 99.3±0.3% in the LD and HD groups, respectively. These results were reliable enough to assess the effects of milk product intake immediately after exercise.

### Dietary intake

Table 4 shows the dietary intake of total energy, protein, carbohydrate, fat, and milks during the training period. The first set of values in the table do not include milk product intake after training, and the second set of values enclosed in the parentheses include milk product intake after training. We confirmed that there were no significant differences in these values among the CNT, LD and HD groups regardless of the milk product intake after training, except for milk and milk products, including supplemental intake, which was significantly higher in the HD group than the CNT group. Moreover, the intake values generally met the recommended dietary allowances for active older Japanese women [31, 32].

### Physical characteristics and fitness

As shown in Table 1, before training, age, height, body weight, BMI, F_EXT_, F_FLX_, and $\dot{V}O_{2\mathrm{peak}}$ were similar among the groups. After training, body weight and BMI decreased in the CNT and LD groups and the $\dot{V}O_{2\mathrm{peak}}$ increased in all groups, but the percent changes in these values did not significantly differ among the groups.

Additionally, blood glucose, triglyceride, high-density lipoprotein cholesterol, low-density lipoprotein cholesterol, and hemoglobin A1c levels before training and the changes after training were similar among the groups (*P* = 0.10–0.77). Furthermore, the fraction of lymphocytes, neutrophils, and monocytes in leukocytes before training and the changes after training were similar among the groups (*P* = 0.078–0.92).

### Thigh muscle strength

Although we did not detect significant differences in the percent increase in F_EXT_ or F_FLX_ among the groups (Table 1), when we analyzed the pattern of increases in muscle strength for our subjects (n = 37), the increase in F_EXT_ and F_FLX_ in each subject was categorized into four patterns: 1) both F_EXT_ and F_FLX_ increased, 2) only F_FLX_ increased, 3) neither increased, and 4) only F_EXT_ increased. We found that 70% of the subjects exhibited an increase in either F_EXT_ or F_FLX_ after training (category 2 + 4), whereas only 30% of the subjects exhibited an increase/decrease in both F_EXT_ and F_FLX_ (category 1 + 3), significantly deviating from the distribution expected by chance (25% for each category) (χ^2^ = 6.1, *P* = 0.014). Therefore, to determine the overall increase in thigh muscle strength, we calculated the average percent increase in F_EXT_ and F_FLX_. The average percent increase was significantly greater in the HD group than the CNT group (Fig 2A). The minimum sample size for detecting the group difference in the average percent increase at α = 0.05 (two-sided) and (1-β) = 0.8, determined using an unpaired t-test, was 14 for each group, which is slightly larger than the sample size in the present study.

### DNA methylation by pyrosequencing

As shown in Table 6, before training, methylation of the *NFKB1* and the *NFKB2* promoter regions at each CpG site was similar among the groups. After training, the percent increase in these values was significantly greater in the HD group than the CNT group, except for in CpG site 1 in *NFKB1*. To determine the overall increase in methylation for each gene, we calculated the average percent increase across CpG sites 1–7 in *NFKB1* and that across CpG sites 1–6 in *NFKB2*. As a result, the average percent increase in *NFKB1* methylation was significantly greater in the HD group than in the other groups (Fig 2B *upper*). Similarly, the average percent increase in *NFKB2* methylation was significantly greater in the HD group than in the other groups (Fig 2B *lower*). The minimum sample size for detecting the group differences in the percent increase in *NFKB1* and *NFKB2* methylation at α = 0.05 (two-sided) and (1-β) = 0.8, determined using an unpaired t-test, was 4 and 6 for each group, respectively, which is smaller than the sample size in the present study. Because methylation of promoter regions is associated with transcriptional suppression of the corresponding gene, enhanced methylation in *NFKB1* and *NFKB2* for the HD group suggests reduced *NFKB1* and *NFKB2* gene expression [33, 34].

### Genome-wide DNA methylation by the Infinium 450K methylation assay

Fig 4A shows typical examples of DNA methylation levels at CpG sites, expressed as normalized β-values with the GenomeStudio Methylation module normalizing algorithm, before vs after training. We observed a high correlation between them both in a CNT subject (*upper* panel) and in a HD subject (*lower* panel); however, when carefully examining the relationships, some CpG sites deviated from the identical lines in both subjects, which suggests that epigenetic changes occurred in some genes after the present intervention.

We detected CpG sites where more profound methylation changes occurred in the HD than in the CNT group via the following two steps. First, we determined the ratio (*Ratio1*) of the β-value after training to that before training in each of the 483,007 CpG sites for the individual subjects, and detected 19,371 DMPs where *Ratio1* values of the HD group were significantly different from those of the CNT group at the level of *P*<0.01 using the Mann-Whitney U test. Second, in every DMP, we chose a median value of the *Ratio1* for each group to determine another ratio (*Ratio2*) of *Ratio1* for the HD group to that for the CNT group. Fig 4B shows the values for *Ratio2* on a base-2 logarithmic scale (*y*) plotted against 19,371 DMPs (*x*), which were ranked by the *Ratio2* values. The number of DMPs on the *x*-axis is an arbitrary unit. The upper and lower areas of the gray lines in the figure indicate the *Ratio1* values of the HD group that were >1.2-fold higher (indicated by red) and lower (indicated by green) than that of the CNT group, respectively, to limit the number of genes used for the following ingenuity pathway analysis (IPA). Using this procedure, we detected 1,448 positions in which more hyper- or hypo-methylation occurred in the HD group than in the CNT group by >1.2-fold.

As shown in Fig 4C, among the 1,448 positions in which more hyper- or hypo-methylation occurred in the HD group than in the CNT group, 45% were located in promoters, and 41% were located within annotated genes (5’ UTR, body, and 3’ UTR regions) (Fig 4C-i). Regarding the location to CpG context, 69% of the more hyper- or hypo-methylated positions were located in CpG-rich regions (CpG islands), and 21% were located in flanking islands (CpG shores) (Fig 4C-ii).

### Ingenuity pathway analysis

To assess the effects of milk product intake during IWT on other inflammatory genes related to the *NFKB* genes, we used an IPA that assisted in systematically understanding the relationships among the genes activated or inactivated by milk product intake during IWT. Among the 1,448 positions in which more hyper- or hypo-methylation occurred in the HD group than in the CNT group by >1.2-fold, the 1,243 positions within gene-associated regions (promoter, 5’ UTR, body, and 3’ UTR) were subjected to IPA because greater changes in DNA methylation within gene-associated regions could have greater effects on gene expression and physiological function.

As a result, we found that the IPA highlighted a cancer-, tumor morphology-, and tissue development-related network as the top-scoring network (score = 42). Additionally, we found that the two highest IPA-ranked canonical pathways were “CREB signaling in neurons” (*P* = 0.00026) and “molecular mechanisms of cancer” (*P* = 0.00040). Furthermore, we found that the three highest IPA-ranked physiological functions were “hematological system development and function” (*P* = 0.0047–0.038), “humoral immune response” (*P* = 0.0047–0.023), and “immune cell trafficking” (*P* = 0.012–0.038). Thus, the highest IPA-ranked networks, pathways, and functions influenced by milk product intake during IWT were associated with inflammatory responses and carcinogenesis.

To visualize the DNA methylation pattern in genes identified by IPA, hierarchical clustering analysis was performed on the dataset, including 83 genes in the top-scoring network, the highest IPA-ranked canonical pathways, and the highest IPA-ranked physiological functions. As shown in Fig 5, the analysis revealed differences in the DNA methylation pattern in the CNT vs the HD group as well as inter-subject variations in methylation.

Fig 6 depicts the top-scoring network derived by the IPA mentioned above, which included several inflammation-related genes, such as *NFKB1*, *MAP3K7*, *JUN*, *TNFSF11*, *TNFRSF11A*, and *MAFB*, which were more hyper-methylated, and *MYC*, which was more hypo-methylated in the HD group than in the CNT group. Because the methylation changes in these genes (except for *TNFRSF11A*) occurred in the promoter regions, the changes could alter the expression of these genes. Indeed, it has been suggested that expression of *MAP3K7*, *JUN*, *TNFSF11* and *MAFB* as well as *NFKB* are epigenetically regulated by DNA methylation [35–37]. Thus, milk product intake during IWT resulted in global methylation changes in inflammation-related genes involving not only NF-κB signaling but also MAP kinase signaling pathways.

## Discussion

The major findings of the present study were that in older women who had performed habitual training prior to this study, post-exercise milk product intake during 5 months of home-based IWT 1) produced a greater percent increase in thigh muscle strength and *NFKB1* and *NFKB2* gene methylation, as determined via pyrosequencing, in a dose-dependent manner and 2) also enhanced global methylation in several inflammation-related genes, as determined via genome-wide DNA methylation analysis.

### Subjects

The BMI, F_EXT_, and F_FLX_ values reported in this study (Table 1) were similar to those previously reported in age-matched female Japanese populations [11, 38, 39], whereas the $\dot{V}O_{2\mathrm{peak}}$ was slightly higher in this study population than other populations, probably because our subjects had performed IWT for more than 6 months before participating in the present study. Thus, the characteristics of the subjects in this study generally reflected those of this age group of the Japanese population.

### Thigh muscle strength

As shown in Fig 2A, HD milk products produced a greater percent increase in thigh muscle strength, which was mainly driven by an increase in F_FLX_ (Table 1).

Regarding the higher response of F_FLX_ to milk product intake during IWT, Nemoto et al. [8] suggested that the sensitivity of F_FLX_ to IWT was greater than that of F_EXT_. Similar results were also reported in other previous studies [15, 40]. Although the precise mechanism is unknown, this might be due to the baseline F_FLX_ value, which was ~50% lower than the F_EXT_ value. According to the current ACSM’s guidelines [6], exercise above a given intensity (80% of the individual’s one repetition maximum) is required to increase muscle strength. Therefore, the exercise intensity during fast walking for IWT was sufficiently high to increase F_FLX_ but not F_EXT_.

In addition, the regional difference in the hypertrophic effects of the training on muscles might be at least partially due to the difference in the landform on which the subjects performed their training. Swanson et al. [22] measured electromyogram amplitude on the knee extensor and flexor muscle groups during running on the treadmill while the slope was varied from 0% to 30% and suggested that the amplitude on the knee extensor muscle group increased as the slope increased, whereas that of the knee flexor muscle group decreased. These results suggest that the effect of IWT on F_EXT_ was enhanced when the subjects walked on inclines, whereas that on F_FLX_ was reduced. Thus, the varied effects of IWT on increasing F_EXT_ and F_FLX_ may be due to the relative exercise intensity compared to their maximal force and/or the varied landform where the subjects performed IWT.

However, it should be noted that milk product intake during IWT likely enhanced the hypertrophic effects on the muscles caused by the training. Despite large variations in training locations among the subjects, we confirmed that there were no significant differences in walking intensity, energy expenditure, or other training variables among the groups (Table 2), and there was a high adherence to the post-exercise milk product intake regimen in both the LD and the HD groups. In addition, there were no significant differences in dietary intake, except milk product supplementation, during the training period among the groups (Table 4). These results suggest that the greater percent increase in thigh muscle strength in the HD group than that of the CNT group was caused by milk product intake during IWT.

Although enhancements in muscle strength elicited by milk protein supplementation have been reported following gym-based resistance training [17], little is known regarding the effects of home-based walking training on muscle strength. We recently reported that supplementation with a mixture of 8 g whey protein and 33 g carbohydrate after every exercise session during 5 months of home-based IWT enhanced the increase in skeletal muscle mass and strength in middle-aged and older women [15]. Consistent with that previous study, we observed a greater percent increase in thigh muscle strength (Fig 2A) by HD milk products that could be obtained more easily than previously used supplements [15, 17] during a 5-month IWT regimen.

We instructed the subjects to consume the milk product(s) within 30 min after daily IWT, as described previously [15, 17]. Kukuljan et al. [41] suggested that daily consumption (but not specifically after exercise) of milk (13 g protein and 22 g carbohydrate) failed to enhance the effects of 18 months of resistance training on muscle strength and mass in middle-aged and older men. In contrast, a study comparing early vs later supplementation after exercise showed that 12 weeks of resistance training increased isokinetic muscle strength and mass in older men who consumed a supplement (10 g of a mixture of milk and soy proteins and 7 g carbohydrate) immediately after exercise but not in those who consumed the same supplement 2 hrs later [18]. These results suggest that milk protein supplementation immediately after every exercise session during IWT is effective at producing the greater percent increase in muscle strength.

Regarding the underlying mechanisms of these effects, Reitelseder et al. [42] examined the effects of a single bolus intake of whey or casein protein immediately after a bout of resistance exercise on muscle protein synthesis rate by administering a continuous infusion of _L_-[1-^13^C]leucine with sequential muscle biopsies to determine the amount of _L_-[1-^13^C]leucine incorporated into muscle protein. They reported that the fractional synthesis rate over 6 hrs after exercise was enhanced by milk protein intake, and that the enhancements elicited by whey and casein intake were similar. Similar results were also reported by others examining the sensitivity of the muscle to milk protein over 5 hrs after resistance exercise [43]. On the other hand, a greater muscle protein synthesis in response to whey than in response to casein was reported over the first 3 hrs after resistance exercise [44]. This result is likely because whey induces a rapid but transient increase in muscle protein synthesis, whereas casein induces a moderate but prolonged response; therefore, whey and casein resulted in similar effects over a longer timeframe [42]. Collectively, these results explain the greater percent increase in muscle strength in the HD group (Fig 2A), whose subjects ingested milk products containing higher amounts of casein (~90%) and lower amounts of whey protein (~10%) than the previous training studies [15, 17]. Additionally, carbohydrate from milk products might also contribute to the greater increase by stimulating insulin secretion, because it has been suggested that insulin, administered with amino acids, stimulates protein synthesis more potently than amino acids alone in human leg tissues [45]. Thus, milk product intake immediately after a bout of exercise likely exerts synergetic effects on the protein synthesis rate in muscle.

In contrast, there have been several studies that have suggested no benefits of post-exercise milk protein intake during prolonged exercise training in regards to enhancing muscle mass or strength [46]. However, these studies were conducted using Western populations, whereas we studied older community-dwelling Japanese subjects, who, in general, have lower milk product intake in their daily life than that of the Western population. For example, milk and milk product intake in Japan is reported to be ~60 kcal/day in middle-aged men [14] and ~150 kcal/day in adolescent females [47], which is similar to the values reported in the present study (Table 4) but is only one-fourth to one-third of those in US and European populations [48–50]. Therefore, the current consensus on the effects of milk protein supplementation based on the results from the Western populations [51] may not be simply applicable to Japanese populations. Considering the lower baseline milk product intake in Japan, it is plausible that post-exercise milk product intake during IWT has more profound effects in Japanese subjects. Importantly, the findings in the present study are consistent with our previous study in older Japanese individuals [15].

### Thigh muscle strength and methylation of pro-inflammatory cytokine genes

As shown in Fig 2B, *NFKB1* and *NFKB2* gene methylation was enhanced by post-exercise milk product intake in proportion to the increase in thigh muscle strength. NF-κB1 and NF-κB2 are family members of NF-κB, which is a well-known transcriptional regulator and plays a central role in inflammation through its ability to induce pro-inflammatory cytokine gene transcription [25]. For example, NF-κB mediates synthesis of cytokines, such as tumor necrosis factor (TNF)-α, interleukin (IL)-1β, IL-6, and IL-8 [25]. Because NF-κB signaling consists of NF-κB1- and NF-κB2-dependent pathways, hyper-methylation of both the *NFKB1* and *NFKB2* gene promoter regions may elicit a reduction in their protein expression, suppressing both pathways and resulting in decreased NF-κB activity and pro-inflammatory cytokine inhibition [52]. This is consistent with the results of large-scale population-based studies showing a close relationship between muscle atrophy with aging and chronic systemic inflammation [2–5], as well as the results of a previous cross-sectional study showing that older men who exercise exhibited increased muscle strength and decreased NF-κB activity compared with that observed in older inactive men [16]. Thus, the enhanced *NFKB* gene methylation observed in the HD group (Fig 2B) suggests that post-exercise milk product intake inhibits pro-inflammatory cytokines by suppressing NF-κB activity. Because we determined DNA methylation using whole blood, the enhanced *NFKB* gene methylation likely represents suppressed inflammation in the whole body, which is likely associated with lessened muscle atrophy in older women.

In addition to the *NFKB* genes, the IPA identified the top network affected by post-exercise milk product intake, including several genes associated with inflammatory responses, such as *MAP3K7*, *JUN*, *TNFSF11*, and *MAFB* (Fig 6), although validation by pyrosequencing was not performed. Enhanced hyper-methylation of these genes by post-exercise milk product intake may suppress MAP kinase signaling, as well as NF-κB signaling pathways [53–56]. On the other hand, enhanced hypo-methylation of *MYC* by post-exercise milk product intake (Fig 6) may be a reaction to the aforementioned hyper-methylation to balance the inflammatory response [57, 58]. Taken together, these results suggest that exercise training combined with milk product intake suppresses not only NF-κB signaling but also other inflammatory signaling pathways, which is consistent with previous observations indicating that exercise training suppresses multiple transcriptomic networks associated with chronic systemic inflammation [59].

In the present study, the highest IPA-ranked networks, pathways and functions affected by post-exercise milk product intake were associated not only with inflammatory responses but also with carcinogenesis. It is well known that persistent, low-grade inflammation causes many LSDs, including cancer [1]. For example, systemic and local increases in the concentrations of inflammatory cytokines stimulate tumor initiation, promotion and progression [1, 60], which are reportedly mediated by the NF-κB signaling pathway [60]. These results suggest that post-exercise milk product intake suppresses chronic and systemic inflammation, thereby protecting against LSDs with an increase in muscle strength.

The precise causal relationship between milk product intake during IWT and the changes in pro-inflammatory cytokine gene methylation remains unclear. However, it has been suggested that chronic systemic inflammation is associated with muscle atrophy with aging [1–5], whereas IWT increases methylation of pro-inflammatory genes [12] and muscle strength [8], and milk protein intake during IWT enhances muscle hypertrophy [15]. In the present study, we found that milk product intake during IWT enhanced methylation of pro-inflammatory genes with increased muscle strength. These results strongly suggest that muscle atrophy with aging is closely associated with pro-inflammatory gene activation, whereas milk product intake during IWT may be a useful countermeasure for prevention.

### Experimental considerations

Three experimental considerations deserve additional discussion. First, we did not measure mRNA, protein expression, or inflammatory markers, such as IL-6, TNF-α, and C-reactive protein [61, 62]. However, based on the results of DNA methylation determined by pyrosequencing and the Infinium 450K methylation assay, we surmised that chronic systemic inflammation is suppressed by post-exercise milk product intake. Additionally, we observed no significant differences in leukocyte fractions during the intervention among the groups, suggesting that the effects of cell composition shifts on DNA methylation changes were minor.

Second, because the subjects in the CNT group were not given a placebo, there is the possibility that those given milk products experienced placebo effects. However, despite a lack of significant differences in training achievements among the groups, we observed dose-dependent effects of milk product intake on the percent increase in muscle strength and *NFKB1* and *NFKB2* methylation (Fig 2), where the measurements were performed by testers who were blinded to subject groupings. Moreover, for the groups that were given milk products, the increase in *NFKB1* and *NFKB2* methylation was significantly greater in the HD group than the LD group (Fig 2B). Therefore, it is unlikely that our findings are related to a placebo effect, although it remains unknown which component of milk products is essential to produce the effects that we observed in the present study.

Third, neither thigh muscle strength nor *NFKB1* and *NFKB2* gene methylation increased in the CNT group (Fig 2), while previous studies reported that ~5 months of IWT elicited an increase in thigh muscle strength [8, 11] and *NFKB2* gene methylation [12]. This may be due to differences in subject baseline conditions because the subjects in the present study had already performed IWT for >6 months prior to the study, whereas those in previous studies had maintained a sedentary lifestyle prior to the studies. In addition, seasonality might also affect changes in *NFKB* gene methylation [63].

## Conclusion

Post-exercise milk product supplementation during home-based IWT enhanced methylation of *NFKB* genes and other pro-inflammatory cytokine genes in conjunction with an increase in thigh muscle strength in middle-aged and older women who had performed habitual training prior to this study.

## Supporting information

S1 Checklist CONSORT checklist (DOC)

S1 Protocol The protocol for the clinical study. (DOCX)

S2 Protocol The protocol for the clinical study in the original language (in Japanese). (PDF)

S3 Protocol The protocol for the genetic study. (DOCX)

S4 Protocol The protocol for the genetic study in the original language (in Japanese). (PDF)
