# Supplementary material for: Effects of milk product intake on thigh muscle strength and NFKB gene methylation during home-based interval walking training in older women: A randomized, controlled pilot study
Source: PLoS One. 2017 May 17;12(5):e0176757. doi: 10.1371/journal.pone.0176757 (PMC5435182; doi:10.1371/journal.pone.0176757)
Supplement: S1 Protocol — (DOCX) [file pone.0176757.s002.docx]

Shinshu University School of Medicine Clinical Study Application Form

January 20, 2012

| 1. Name of plan | | | Effects of milk product intake on improvements of QOL and physical fitness in elderly persons | | | |
| --- | --- | --- | --- | --- | --- | --- |
| 2. Classification of study organizations | | | [1] Study by a single group | | | |
| 3. Wishes for inspection | (1) Inspection method to be expected | | [2] Expedited review (→ Descriptions in (2) are necessary) | | | |
|  | (2) Reasons for expedited review | | [1] This is a collaborative clinical study, and its protocol was approved by the Ethics Review Board of the primary institution.  (Note. Please attach a certificate of approval by the Ethics Review Board of the primary institution)  Minor changes of “Plasma protein-increasing mechanism related to exercise training in elderly persons and effects of protein supplement consumption on the mechanism”(Approval No.: 1769), which was approved by the Ethics Review Board of the School of Medicine.  The following points were changed:  1) With respect to training methods, bicycle exercise training was changed to interval walking training.  2) The training period was changed from 2 to 5 months.  3) Carbohydrate/protein supplements were switched to yoghurt/cheese.  4) Concerning measurement items, glucose tolerance, bone mineral density, and inflammatory gene methylation were added, and carotid compliance and 24-hour blood pressure were deleted.  5) “Measurement of inflammatory gene methylation” was approved by the Human Gene Analysis Ethics Review Board (“Verification of the preventive effects of the combination of exercise and supplement consumption on lifestyle-related diseases---Investigation of blood cell DNA methylation and its verification from the viewpoint of epigenetics”(Receipt No.: 337)).  [2] Cases in which there are no risks exceeding minimized ones for subjects (socially acceptable risks that do not exceed the potential limits of physical, mental, or social damages related to daily living or routine medical examinations) | | | |
| 4. Study classification | | | [2] Study with intervention (excluding those corresponding to [1]) | | | |
| 5. Ethical guidelines to be referred | | | Ethical guidelines regarding clinical studies | | | |
| 6. Necessity of database registration | | | [1] Necessary. | | | |
| 7. Disclosure of the results of inspection by the Ethics Review Board | | | [1] The above information may be disclosed. | | | |
| 8. Institution/place | | | Department of Sports Medical Sciences, Shinshu University Graduate School of Medicine | | | |
| 9. Chief investigator (affiliation/managerial position) | | | Name:  Hiroshi Nose | Affiliation:  Department of Sports Medical Sciences, Shinshu University Graduate School of Medicine | Managerial position:  Professor | Date of educational training (lecture or audiovisual education)  (February 18, 2009) |
| 10. Investigators (affiliation/managerial position)  Note) If the study is conducted in cooperation with affiliated hospitals，at least 1 person responsible for the study in each hospital should be described as an investigator. | | | Name:  Yoshi-ichiro Kamijo | Affiliation:  Department of Sports Medical Sciences, Shinshu University Graduate School of Medicine | Managerial position:  Assistant professor | Date of educational training (lecture or audiovisual education)  (February 18, 2009) |
| 11. Request for clinical study coordinators’ support | | | [2] No request. | | | |
| 12. Collaborative institution (Role)  Note) The role of each institution should be described in a parenthesis. | | |  | | | |
| 13. Subjects | (1) Number of subjects (plan) | | 60 | | | |
|  | (2) Selection criteria | | 1) Persons aged 50 to 75 years who had participated in the “Matsumoto Jukunen Taiikudaigaku” for 6 months or more (continuing members)  2) Those with a systolic blood pressure of ≤159 mmHg and a diastolic blood pressure of ≤99 mmHg (for safety assurance)  3) Those without a medical history influencing interval walking training  4) Those who wished to participate in this study on recruitment | | | |
|  | (3) Presence or absence of persons aged <20 years, adults with impaired judgment, or dead persons | | [4] No such subject is included (→ No description in No. 22 is necessary.) | | | |
| 14. Significance/purpose of this study | | | This study follows studies approved by the Ethics Review Board (Receipt Nos.: 385 and 1769). We indicated that carbohydrate/protein supplement consumption during exercise training increased the plasma volume by promoting albumin synthesis in the liver, improving the body temperature-controlling capacity, muscle strength, and cardiopulmonary function. In addition, we reported that symptoms of lifestyle-related diseases, such as hypertension and hyperglycemia, decreased with improvements in the muscle strength and cardiopulmonary function.  On the other hand, according to recent studies, atrophy of the skeletal muscle related to aging or lack of exercise induces the secretion of several bad cytokines from muscles, and they cause chronic systemic inflammation, leading to arteriosclerosis through blood vessels, diabetes through the pancreas, and depression/dementia through the brain. In contrast, an exercise-related increase in the muscle volume inhibits the secretion of these cytokines (or promotes the secretion of good cytokines), reducing symptoms of lifestyle-related diseases. Our findings described above support this hypothesis. However, carbohydrate/protein supplements are expensive, and people feel inconvenient when purchasing them.  The purpose of this study is to verify the experimental hypothesis that the consumption of yoghurt/cheese (containing components similar to those of carbohydrate/protein supplements) during interval walking training for 5 months may more markedly improve the aerobic capacity, muscle strength, glucose tolerance, body temperature-controlling capacity, and lifestyle-related diseases in elderly persons, as demonstrated for the above supplements. | | | |
| 15. Study methods | | | [Study protocol]  In April, all subjects will be requested to gather at a gymnasium for morphological measurement, physical strength measurement, and hematology. For physical strength measurement, endurance and lower limb muscle strength will be measured. For a blood test, items as parameters of lifestyle-related diseases will be measured. On another day, the subjects will be requested to go to the department of sports medical sciences, Shinshu University for measurement of the blood volume and body temperature-controlling capacity and a glucose tolerance test. In addition, the bone mineral density will be determined on another day.  Subsequently, the subjects will be randomly divided into 3 groups: control (CNT group, n=15), low-milk-product-treated (LMT group, n=15), and high-milk-product-treated (HMT group, n=15) groups based on the above measurements so that there are no differences in physical properties.  Subsequently, interval walking training for 30 minutes or more on 4 days or more per week will be conducted for 5 months. During this period, the training volume will be recorded using a portable caloric meter and Internet-based remote-type individualized exercise prescription system. During training, in the LMT group, the subjects will be instructed to consume 1 unit* of yoghurt or cheese containing 4 g of protein alternately within 30 minutes after exercise on a day when fast walking is achieved for 15 minutes or more. In the HMT group, the subjects will be instructed to consume 2 units of yoghurt and 1 unit of cheese so that protein intake is 12 g. Furthermore, all groups will be prohibited from eating a meal within at least 1 hour after the completion of fast walking. In addition, a survey on diet during this period will be conducted. In the milk-product-treated groups, the subjects will be instructed to avoid snacks so that there may be no differences in total energy intake among the 3 groups. Samples will be collected from May 1, and mid-phase physical strength measurement will be performed in Week 10.  The subjects will gather again at a gymnasium or the department of Sports Medical Sciences, Shinshu University, from 20 weeks after the start of training (late September), and the effects of milk product consumption will be evaluated through measurements similar to those before training.  * One portion of commercially available low-fat yoghurt (112 g, protein content: 4.1 g) or cheese (80 g, protein content: 4.1 g) is regarded as 1 unit.  [Measurement item]  [1] Physical strength measurement: Endurance (3-step loading method), muscle strength (measurement of isometric muscle strength, GT330)  [2] Morphological measurement: Height, body weight, %FAT, abdominal circumference, blood pressure  [3] Hematology: Hct, Hb concentration, albumin concentration, total cholesterol concentration, HDL, LDL, fasting blood sugar, HbA1c, uric acid  [4] Measurement of inflammatory gene methylation: Measurement of the methylation of 5 genes associated with the onset of lifestyle-related diseases, cancer, or depression (ASC, Adiponectin, Leptin, BDNF, and NF-kB)(concerning the consent form, the subjects will be requested to sign another informed consent form with respect to the contents approved by the Human Gene Analysis Ethics Committee (Receipt No. 337).)  [5] Measurement of the blood volume: Pigment dilution method with Evans Blue  [6] Measurement of the body temperature: After blood volume measurement, the subjects will be instructed to wear a bathrobe and outfit for cold weather. After confirming a constant body temperature in a sitting position at rest, foot heating will be conducted using a foot warmer. During this period, the tympanic membrane temperature and sweat rate must be measured. In addition, the skin blood flow will be determined using a laser Doppler blood flowmeter.  [7] Glucose tolerance test: After measuring the body temperature-controlling capacity and blood volume, a glucose tolerance test will be conducted. After 75-g glucose loading, blood will be collected every 30 minutes for 3 hours to measure the blood glucose, insulin, serum creatinine, Hct, Hb, and plasma electrolyte levels. In addition, urine will be collected every 30 minutes to measure the urine volume and urinary creatinine level. The subjects will be instructed to drink 100 mL of water after urine collection.  [8] Bone mineral density: The subjects will be requested to consult the Department of Radiology, Shinshu University, and femoral and lumbar vertebral bone mineral density will be measured using DEXA. | | | |
| 16. Study period (maximum: 5 years) | | | Start: [1] from the day of approval  Completion: March 31, 2017 | | | |
| 17. Advantages and disadvantages related to study participation | | (1) Advantages related to study participation | [2] Direct advantages may be obtained (the combination of exercise training and yoghurt/cheese consumption may improve the physical strength and body temperature-controlling capacity, and reduce symptoms of lifestyle-related diseases.). | | | |
|  |  | (2) Future advantages related to the outcome of this study | Our method may contribute to nursing and the development of exercise/diet therapies for the prevention of lifestyle-related diseases or heatstroke. | | | |
|  |  | (3) Risks/discomfort related to study participation | 1) Measurement of the plasma volume  A blue pigment, Evans B1ue, will be used. Evans B1ue adheres to blood proteins, such as albumin, retaining in blood for a specific period after measurement. However, it is excreted within 2 to 3 weeks. This pigment was developed at the NASA, and it is nonradioactive and safe. Before its use, the presence or absence of allergy must be confirmed. However, if shock or intoxication symptoms appear during its use, appropriate treatment should be promptly performed in cooperation with affiliated hospitals. This measurement method was approved (Receipt No.: 385).  2) Others  Interval walking training may induce muscular pain or arthralgia, and yoghurt/cheese consumption may cause diarrhea in the initial phase, although these symptoms are rare. If physicians consider treatment necessary due to severe symptoms, appropriate treatment should be promptly performed in cooperation with affiliated hospitals. | | | |
| 18.Storage of samples | | (1) Presence or absence of sample storage during the study period | [1] Stored in the institution (→it is necessary to describe the following columns) | | | |
|  |  | (2) Storage methods and period ((1) = [1] or [2]) | [2] Stored as data (storage period: until March 31, 2017) | | | |
|  |  | (3) Manager responsible for sample storage | [1] Chief investigator of our university | | | |
|  |  | (4) Presence or absence of sample storage after the completion of this study | [1] Stored in the institution (place: Department of Sports Medical Sciences, Shinshu University Graduate School of Medicine) | | | |
|  |  | (5) Necessity of storage/reasons for storage ((4) = [1] or [2]) | [1] To utilize samples/materials for future studies  (expected study contents: ) | | | |
|  |  | (6) Type of anonymization ((4) =[1] or [2]) | [2] Linkable anonymization | | | |
|  |  | (7) Sample-discarding method | [2] Burning with anonymization | | | |
| 19. Publication of the study outcome | | | [1] The results will be published at society meetings or on academic journals after anonymizing the subjects. | | | |
| 20. Protection of personal information regarding this study  Note: Please write this in reference to explanations at the end of this application form | | (1) Personal information-protecting method | [2] Linkable anonymization | | | |
|  |  | (2) Anonymization method ((1) = [2] or [3]) | Name coding. When analyzing the data after the completion of this study, only name codes will be used. | | | |
|  |  | (3) Name (affiliation) of a person managing personal information((1) = [2] or [3]) | Keiichi Higuchi (Professor, Graduate School of Medicine, Shinshu University) | | | |
|  |  | (4) Correspondence table-managing method in the case of linkable anonymization ((1) = [2]) | [1] Computers separated from other computers must be used. The data should be recorded in an external memory medium, key-locked, and strictly stored. | | | |
| 21.Procedures for obtaining informed consent | | | [1] Written informed consent should be obtained from each subject prior to this study.  (It is unnecessary to describe Nos. 22 and 24. →Skip to No. 23 ) | | | |
| 22. Cases in which informed consent is obtained from alternatives (21 = [2])  Note: 21 = [1] or [3] or [4]: It is unnecessary to describe this column.  (13 (3) = [1] or [2] or [3]: if corresponding to 21 = [4], it is unnecessary to describe this column) | | | Not applicable. | | | |
| 23. Matters to be described in the explanatory document for obtaining informed consent (21 = [1] or [2])  Note. Please attach the explanatory document and informed consent form. | | | [1] Study participation is voluntary.  [2] Even if the subject does not consent to study participation, there may be no disadvantage.  [3] The subject or alternative may withdraw from this study without disadvantages even after signing informed consent.  [4] Reasons for subject selection  [5] Significance, purpose, methods, and period of this study  [6] Name and affiliations of investigators  [7] Expected study results, study participation-related advantages, risks/discomfort, and management after the completion of this study  [8] The subject or alternative can obtain or inspect materials on the study plan and methods based on their wishes if personal information on other subjects or study originality is protected.  [9] Handling of personal information (If personal information is provided to another institution, the institution’s name and purpose must be examined by the Ethics Review Board before providing the study results.)  [10] Storage/use of samples and storage period  [11] Possibility that the study outcome may lead to a patent and organization to which the patent will belong  [12] Possibility that the study outcome may be published with anonymization  [13] Source of funds regarding this study, relationship of investigators with companies/corporations, and conflict of interest  [14] Compensation for health damage (When there is a study-related compensation, the contents of the compensation are included.)  [15] Information on the inquiry/complaint counter | | | |
| 24. Cases in which this study will be conducted without obtaining informed consent  Note: If informed consent is obtained from the subject or alternative prior to this study (21 = [1] or [2] or [3]), it is unnecessary to describe this column. | | (1) Subjects | Not applicable. | | | |
|  |  | (2) (1) = [2]: Type of materials and data  Note: (1) = [1]: It is unnecessary to describe the column. | Not applicable. | | | |
|  |  | (3) Study properties | Not applicable. | | | |
|  |  | (4) Countermeasures replacing informed consent | Not applicable. | | | |
|  |  | (5) (4) = [1]: Contents of countermeasures replacing informed consent | Not applicable. | | | |
| 25. Utilization of provided samples | | (1) Presence or absence of the utilization of provided samples | [2] No use. | | | |
|  |  | (2) Presence or absence of a consent if the sample is utilized (1= [1]) | Not applicable. | | | |
|  |  | (3) Sample-anonymizing method in the absence of a consent (2=②) | Not applicable. | | | |
|  |  | (4) Linkable anonymization with a correspondence table in the absence of informed consent (3 = [3]) | Not applicable. | | | |
| 26. Funds regarding this study | | | [4] Scholarship contributions/grants  [6] Others (provided by NPO Corporation/Jukunen Taiikudaigaku Research Center) | | | |
| 27. Relationship of this study with companies/corporations | | | [1] No company will be involved in this study. | | | |
| 28. Conflict of interest^11^  (Check all corresponding items.)  Note: This item is established to adequately manage information disclosure-related conflict of interest. Even if the situation corresponds to this, there may be no need to change the study plan when it is within the permissible range of our university (for study promotion/protection of investigators).  Please describe this in reference to explanations at the end of this application form.  However, matters regarding this item will be examined according to the “Shinshu University Regulations for Conflict-of-Interest Management regarding Clinical Research, if necessary. | | | [6] There may be no conflict of interest. | | | |
| 29. Compensation for adverse events, such as health damage, related to this study | | | [4] Health insurance-based management | | | |
| 30. Confirmation matters prior to this study  (Please check all corresponding items) | | | [1] The chief investigator has sufficient special knowledge and clinical experience necessary for adequately conducting this study.  [2] The chief investigator must promptly notify the development of serious adverse events or disturbance related to the clinical study to the director of each institution.  [3] The chief investigator must discontinue or complete this study if risks exceed expected advantages related to this study or when sufficient outcomes are obtained. Furthermore, the contents should be promptly reported to the director of each institution.  [4] The chief investigator must understand information on this study, such as presentations at society meetings and publications in Japan and other countries (publication information), until the completion of this study, and report the publication information to the director of each institution.  [5] The chief investigator must report the status of study progression once a year or the results at the completion of this study in writing to the director of each institution.  [6] The chief investigator must take countermeasures necessary for the protection of personal information prior to the start of this study.  [7] The chief investigator must make efforts even after the completion of this study so that the subjects may receive the best prevention, diagnosis, and treatment obtained based on the results of this study.  [8] Investigators must receive training/education regarding knowledge necessary for conducting clinical research prior to the clinical study.  [9] Investigators must conduct research based on the scientific literature, other sources of information regarding science, and sufficient experiments according to acceptable scientific rules.  [10] Investigators must publish the study results so that the subjects may not be identified.  [Studies with health-affecting behavior involving humans (interventional studies)]  [11] It is necessary to obtain adequate advices from physicians with sufficient clinical experience (if the physician is not included in the study team, the physician’s name should be described) | | | |
| 31. Other matters to be mentioned | | |  | | | |
