# Supplementary material for: Effects of milk product intake on thigh muscle strength and NFKB gene methylation during home-based interval walking training in older women: A randomized, controlled pilot study
Source: PLoS One. 2017 May 17;12(5):e0176757. doi: 10.1371/journal.pone.0176757 (PMC5435182; doi:10.1371/journal.pone.0176757)
Supplement: S2 Protocol — (PDF) [file pone.0176757.s003.pdf]

3

（信州大医臨床研究計画様式１）

信州大学医学部臨床研究計画申請書

提出日	平成24年　1月　20日
提出番号＊	
諾否決定日＊	
諾否＊	
承認番号＊	

＊申請者においては記入しないこと

以下，項目番号を変更しないこと；朱書きの注意書きを削除しないこと.

1. 計画名		乳製品の摂取による高齢者の QOL 向上および運動能力改善効果の検討
2. 研究組織の分類		■ ① 単独グループによる研究<br>□ ② 多施設共同研究の主任研究者<br>□ ③ 多施設共同研究への参加（主任施設名：　　　　）<br>（他施設の倫理委員会による承認証明書の添付：□あり　□なし）<br>□ ④ その他（　　　　　　　　　）
3. 審査希望	(1) 希望する審査方法	□ ①一般審査<br>■ ②迅速審査（→（2）への記載が必要）
	(2) 迅速審査理由	■ ① 共同研究であって，既に主任研究施設において倫理委員会の承認を受けた臨床研究計画であるため<br>（注：主任研究施設の倫理委員会による承認証明書を添付すること）<br><br>すでに医倫理委員会で承認されている「高齢者の運動トレーニングによる血漿蛋白質増加機構並びにそれに及ぼすタンパク室サプリメント摂取の効果」（承認番号：1769）の軽微な変更。<br>主な、変更点は以下のとおり、<br>1）トレーニング方法について、自転車運動トレーニングから、インターバル速歩トレーニングに変更した。<br>2）トレーニング期間について、2ヵ月から5ヵ月に変更した。<br>3）サプリメントについて、糖質・蛋白質サプリメントからヨーグルト・チーズへ変更した。<br>4）測定項目について、耐糖能測定、骨密度測定、炎症性遺伝子のメチル化測定を追加し、頸動脈コンプライアンス測定、24時間血圧測定を削除した。<br>5）「炎症性遺伝子のメチル化測定」については、ヒト遺伝子解析倫理委員会の承認を得ている（「運動と補助食品摂取の併用による生活習慣病予防効果の検証ー血液細胞の DNA メチル化を調べそのエピジェネティックからの検証ー」（受付番号 337））。<br><br>■ ② 被験者に対して最小限の危険（日常生活や日常的な医学検査で被る身体的，心理的，社会的危害の可能性の限度を超えない危険であって，社会的に許容される種類のものをいう。）を超える危険を含まない場合<br>□ ③ 被験者の意思に回答が委ねられている調査であって，その質問内容により被験者の心理的苦痛をもたらすことが想定されないものである場合。<br>□ ④ 研究者等が所属する医療機関内の患者の診療録等の診療情報を用いて，専ら集計，単純な統計処理等を行う研究である場合

4

	□ ⑤ 次に掲げる事項についての規程を含む契約に基づき，データの集積又は統計処理のみを受託する場合<br>ア．データの安全管理<br>イ．守秘義務			
4. 研究の分類	□ ① 介入を伴う研究であって，医薬品又は医療機器を用いた予防，診断又は治療方法に関するもの<br>■ ② 介入を伴う研究（①に該当するものを除く。）<br>□ ③ 観察研究［介入を伴わず試料等を用いた研究であって，疫学研究（明確に特定された人間集団の中で出現する健康に関する様々な事象の頻度及び分布並びにそれらに影響を与える要因を明らかにする科学研究をいう。）を含まないもの］<br>□ ④ 疫学研究			
5. 参照すべき倫理指針	■ ① 「臨床研究に関する倫理指針」<br>□ ② 「疫学研究に関する倫理指針」<br>□ ③ なし（理由：　）			
6. データベースへの登録の必要性	※臨床研究に係る倫理指針において，4 が①又は②の研究であり，侵襲性を有するものはデータベースへの登録が義務付けられています.（→項目4＝①であり，項目6＝①の場合は項目29(1)①の補償の措置を講じること.）<br>（登録先：UMIN臨床試験登録システム（国立大学附属病院長会議））<br>■ ① 登録する必要有り<br>□ ② 登録する必要なし<br>理由　□ 介入研究ではないため<br>□ その他（　）			
7. 倫理委員会審査結果の公開	※臨床研究に関する倫理指針において，倫理委員会審査の会議の記録の概要を公表することが定められています。下記の項目を委員会のホームページで公開します.<br>1. 研究計画名<br>2. 研究者氏名（申請者・委員会当日の説明者）<br>3. 審査結果<br>■ ① 上記の情報のまま公開可<br>□ ② 上記の情報のままでは公開不可のため，以下のように訂正を求める<br>（訂正内容を具体的に記載：　）			
8. 研究機関名・研究実施場所	医学系研究科加齢適応医科学系専攻 個体機能学部門 スポーツ医科学分野<br>加齢適応医科学棟 人工気象室			
9. 研究実施責任者氏名（所属・職名）	氏名<br>能勢 博	所属<br>医学系研究科 加齢適応医科学系専攻 個体機能学部門 スポーツ医科学分野	職名<br>教授	□教育研修受講日または視聴日<br>(H21 年 2月 18日)
10. 研究者等氏名（所属・職名）<br><br>注）関連病院の協力を得て行う研究の場合は，各病院で研究に関わる者を少なくとも1名研究者として記載する.	氏名<br>上條 義一郎	所属<br>スポーツ医科学	職名<br>助教	□教育研修受講日または視聴日<br>(H21 年 2月 18日)
				□教育研修受講日または視聴日<br>（ 年 月 日）
				□教育研修受講日または視聴日<br>（ 年 月 日）
				□教育研修受講日または視聴日<br>（ 年 月 日）

				□教育研修受講日または視聴日<br>（　年　月　日）
				□教育研修受講日または視聴日<br>（　年　月　日）
				□教育研修受講日または視聴日<br>（　年　月　日）
				□教育研修受講日または視聴日<br>（　年　月　日）

11. 臨床研究コーディネーターの支援の要請		□ ① 支援を要請する<br>■ ② 支援を要請しない
12. 共同研究機関名（役割）<br><br>注）各機関の役割をカッコ書きで記載する.		
13. 被験者	(1) 被験者数（予定）	60 人
	(2) 被験者の選定方針	1)「松本熟年体育大学」事業に半年以上参加されている 50－75 歳の一般中高年の方(継続会員)<br>2)安全の確保のために、収縮期血圧が 159mmHg 以下、かつ拡張期血圧が 99mmHg 以下の方<br>3)インターバル速歩トレーニングに支障をきたす既往症がない方<br>4)参加募集に対して自主的に参加を希望された方
	(3) 被験者に未成年者，判断能力の不十分な成年者，または死者が含まれるか	□ ① 未成年者（→22 への記載が必要）<br>□ ② 判断能力の不十分な成年者（→22 への記載が必要）<br>□ ③ 死者（→22 への記載が必要）<br>■ ④ ①－③の者は含まれない（→22 への記載は不要）
14. 研究の意義・目的		本研究は、倫理審査の承認(受付番号 385 と 1769)を既に受けた研究の継続研究である。我々は運動トレーニング中の糖質・蛋白質補助食品摂取が肝臓のアルブミン合成を促進することで血漿量を増加させ、体温調節能、筋力、心肺機能の向上に効果があることを明らかにした。さらに、筋力、心肺機能の向上に比例して、高血圧、高血糖などの生活習慣病の症状が改善することを明らかにした。<br>一方、最近の報告によれば、加齢や運動不足による骨格筋の委縮は、筋肉からの複数の悪玉サイトカインの分泌をおこし、それらは全身の慢性炎症を引き起こし、血管に働くと動脈硬化、膵臓に働くと糖尿病、脳に働くと「うつ」や「認知症」を発症させるが、逆に、運動によって筋肉量が増加すると、それらのサイトカインの分泌を抑制し(あるいは、善玉サイトカインが分泌され)生活習慣病の症状を改善することが明らかになっている。上記の我々の得た所見は、この説を支持する結果となっている。しかし、上記で用いた糖質・蛋白質補助食品は一般に高価で購入するのも不便であることが弱点となっていた。<br>そこで、本研究の目的は、高齢者において 5 カ月間のインターバル速歩トレーニング中に、(糖質・タンパク質サプリメントと同様の成分である)ヨーグルト・チーズを摂取することによって、同サプリメント摂取時と同様に好気的運動能、筋力、耐糖能、体温調節能、生活習慣病の改善がより亢進するのではないか、という実験仮説を検証することである。
15. 研究方法		【研究プロトコール】<br>4 月に、被験者全員に体育館に集まっていただき、形態測定、体力測定、

血液検査を行う。「体力測定」は、持久力、下肢筋力を測定する。「血液検査」は一般の生活習慣病指標となる項目を測定する。また、別の日に、信州大学スポーツ医科学分野に来ていただき、「血液量測定」、「体温調節能測定」と「糖負荷試験」を行う。さらに、別の日に「骨密度測定」を行う。

その後、上記の測定値にしたがって、被験者を身体特性に差がないように、無作為にコントロール群(CNT群、15名)、低乳製品摂取群（LMT群、15名)、高乳製品摂取群(HMT群、15名)の3群に分ける。

その後、>4日/週、>30分間のインターバル速歩トレーニングを5ヶ月間実施し、その間の「トレーニング量」を携帯型カロリー計とインターネットを利用した遠隔型個別運動処方システムによって記録する。トレーニング中、LMT群においては速歩を15分以上達成した日の運動後30分以内に蛋白質4gを含有するヨーグルトあるいはチーズを1単位*を交互に、一方、HMT群においては、蛋白質12gとなるように、ヨーグルト2単位＋チーズ１単位を摂取してもらう。また、全群において速歩終了後少なくとも1時間は食事を控えていただく。さらに、この間の「食事調査」を実施するが、乳製品摂取群については、その分、間食を控えていただき、総エネルギー摂取量が群間で差がでないようにしていただく。試料摂取は5月1日からとし、10週目に「中間期体力測定」を行う。

トレーニング開始ご20週目の9月下旬から、再度、体育館、信州大学スポーツ医科学分野に集合していただき、トレーニング前と同様の測定を行い乳製品の摂取効果を判定する。

*市販の低脂肪ヨーグルト112g (蛋白含有量4.1g) あるいは市販のチーズ 80g (蛋白含有量 4.1g)をそれぞれ1個を1単位とする。

【測定項目】
① 体力測定:持久力(3段階負荷漸増法)、筋力(等尺性筋力測定、GT330)
② 形態測定:身長、体重、%FAT、腹部周囲径、血圧
③ 血液検査:Hct、Hb 濃度、アルブミン濃度、総コレステロール濃度、HDL、LDL、空腹時血糖、HbA1C、尿酸
④ 炎症性遺伝子のメチル化測定:生活習慣病、がん、うつ発症に関連する5個の遺伝子 (ASC, Adiponectin, Leptin, BDNF, NF-kBなど) について、そのメチル化の測定 (この承諾書については、ヒト遺伝子解析倫理委員会で承認された内容について(受付番号337)別途同意書に署名をお願いする)
⑤ 血液量測定:Evans Blueによる色素希釈法測定
⑥ 体温測定:血液量測定後、バスローブ、防寒具を着ていただき、座位安静にて体温が一定になるのを確認した後、足温器に足をつけヒーティングを行う。その間、鼓膜温と発汗速度を測定する。さらにレーザードップラー血流計で皮膚血流量を測定する。
⑦ 糖負荷試験:体温調節能の測定後、血液量測定に続き、糖負荷試験を行う。75gの糖負荷後、30分毎、3時間にわたり血液を採取し、血糖、インシュリン、血清クレアチニン、Hct、Hb、血漿電解質を測定する。さらに、30分毎に尿を採集し、尿量と尿中クレアチニン濃度を測定する。また、採尿後 100mlの真水を摂取させる。
⑧ 骨密度:信州大学の放射線科に来院していただき、大腿骨、腰椎の骨密度をDEXA法にて測定を行う。

16. 研究期間（最大５年）	開始：■ ①承認日より<br>②<br>終了：　H29　年　3　月　31日まで

7

17. 研究参加に伴う利益および不利益	(1) 研究に参加することにより被験者にもたらされうる利益	□ ① 直接的な利益は期待できない<br>■ ② 直接的な利益が期待できる（具体的に：運動トレーニングとヨーグルト、チーズの摂取を併用することにより、体力向上ばかりでなく、体温調節能の改善、生活習慣病症状の改善が期待できる。）
	(2) 研究の成果によって将来的にもたらされうる利益	介護・生活習慣病予防、熱中症予防ための運動・食事療法の開発につながる。
	(3) 研究に参加することにより被験者に起こりうる危険・研究に伴う不快な状態	1) 血漿量の測定<br>青色の色素 Evans Blue を使用する。Evans Blue は、血中のアルブミンなどのタンパク質に付着するため、測定後しばらくは血中に残るが、2〜3週間ほどで体外へ排泄される。NASA で開発された色素であり、非放射性で安全性は高い。色素使用に際して、事前にアレルギーの有無を十分確認する。しかし、万が一使用中にショックおよび中毒症状が現れた場合は、直ちに付属病院との連携により適切な処置を行う。この測定方法は、既に承認(受付番号 385)受けたものである。<br><br>2) その他<br>非常に稀だが、インターバル速歩トレーニングに伴い筋肉痛、関節痛が生じる可能性、ヨーグルト・チーズ摂取により当初下痢をする可能性があるが、それらの症状がひどく、医師の診断の結果、治療が必要と判断された場合は、直ちに付属病院との連携により適切な処置を行う。
18. 試料等の保存について	(1) 研究期間中の試料等の保存の有無	■ ① 機関内で保存する（→以下への記載が必要）<br>□ ② 共同研究機関で保存する（→以下への記載が必要）<br>□ ③ 保存しない　（→（7）への記載が必要）
	(2) 保存する場合（(1)＝①または②），その方法と保存期間	□ ① 血液・組織等のまま保存（保存期間　　年　月　日まで）<br>■ ② データとして保存（保存期間　H29　年　3　月　31 日まで）<br>□ ③ その他（　　　　　　　）
	(3) 試料等の保存の管理責任者	■ ① 本学の研究実施責任者<br>□ ② ①以外の研究者が管理する（→管理責任者氏名を記載）<br>所属：<br>職名：<br>氏名：
	(4) 研究終了後の試料等の保存の有無	■ ① 機関内で保存する（場所：医学系研究科･スポーツ医科学分野）<br>□ ② 共同研究機関で保存する<br>□ ③ 保存しない
	(5) 保存する場合（(4)＝①または②），その必要性・理由	■ ① 将来の研究ための貴重な試料・資料とするため<br>（予測される研究内容：　　　　　　　）<br>□ ② その他（　　　　　　　）
	(6) 保存の場合（(4)＝①または②），匿名化の種類	□ ① 連結不可能匿名化<br>■ ② 連結可能匿名化
	(7) 試料等を廃棄する際の方法	□ ① 匿名化のまま，密封容器に廃棄<br>■ ② 匿名化のまま，焼却処分<br>□ ③ 匿名化のまま，紙データについてはシュレッダー等を用いて廃棄し，電子データについてはデータを完全に消去する<br>□ ④ その他（　　　　　　　）
19. 研究成果の公表		■ ① 被験者を特定できないようにした上で，学会や学術雑誌で公表する<br>□ ② 予定していない<br>□ ③ その他（　　　　　　　）

20. 研究に係る個人情報[i]の保護<br>注：本申請書末尾の解説を参照の上，具体的に記載する．	(1) 個人情報保護の方法	□ ① 匿名化しない<br>■ ② 連結可能匿名化<br>□ ③ 連結不可能匿名化<br>□ ④ その他（　　　　　　　　　　）
	(2) 匿名化を行う場合（(1) ＝②または③)，具体的な匿名化の方法	被験者を氏名コード化する。研究終了後、データの解析を行う際には、氏名コードのみを用いる。
	(3) 匿名化を行う場合（(1) ＝②または③)，個人情報管理者氏名（所属・職名）	樋口京一(信州大学大学院医学系研究科　教授)
	(4) 連結可能匿名化を行う場合（(1) ＝②)，対応表の管理方法	■ ① 他のコンピュータと切り離されたコンピュータを使用し，外部記憶媒体に記録させ，その記憶媒体は，鍵をかけて厳重に保管する．<br>□ ② 筆記による原簿として，鍵をかけて厳重に保管する．<br>□ ③ その他（具体的に：　　　　　　　　）
21. インフォームド・コンセントのための手続		■ ① 被験者からインフォームド・コンセントを文書で得て研究を行う（→22，24への記載は不要，→23へ）<br>□ ② 代諾者等からインフォームド・コンセントを文書で得て研究を行う（→22に必ず記載のこと，→23へ）<br>□ ③ 観察研究のうち試料等の採取が侵襲性を有しない研究であり，文書による同意に代えて，説明内容及び被験者から受けた同意に関する記録を診療録等に記載して研究を行う（→原則として研究概要と23の内容を口頭で説明し，同意を得た年月日を診療録等に記載すること）<br>□ ④ インフォームド・コンセントを得ないで研究を行う（→24に必ず記載する）<br>□ ⑤ その他（　　　　　　　　　　）
22. 代諾者等からインフォームド・コンセントを得て研究を行う場合（21＝②）<br>注：21＝①または③または④の場合は本欄への記載不要．（13 (3) ＝①または②または③であっても，21＝④であれば，本欄への記載不要．）		[当該研究の重要性]<br><br>[被験者の参加が研究を実施するに当たり必要不可欠な理由]<br><br>[代諾者等の選定方針]
23. インフォームド・コンセントを得るための説明文書記載事項（21＝①または②）<br>注：説明文書と同意書を必ず別添．		■ ① 研究への参加は任意であること<br>■ ② 研究への参加に同意しないことをもって不利益な対応を受けないこと<br>■ ③ 被験者又は代諾者等は，自らが与えたインフォームド・コンセントについて，いつでも不利益を受けることなく撤回できること<br>■ ④ 被験者として選定された理由<br>■ ⑤ 研究の意義，目的，方法及び期間<br>■ ⑥ 研究者等の氏名及び職名<br>■ ⑦ 予測される研究の結果，研究に参加することにより期待される利益及び起こりうる危険並びに必然的に伴う不快な状態，研究終了後の対応

		■ ⑧ 被験者及び代諾者等の希望により，他の被験者の個人情報保護や研究の独創性の確保に支障がない範囲内で，研究計画及び研究の方法についての資料を入手又は閲覧することができること<br>■ ⑨ 個人情報の取扱い（個人情報を他の機関に提供する可能性がある場合は，提供先の機関名，提供先における利用目的が妥当であること等について倫理審査委員会で審査した上で研究の結果を提供することを含む.）<br>■ ⑩ 試料等の保存及び使用方法並びに保存期間<br>■ ⑪ 研究の成果により特許権等が生み出される可能性があること及び特許権等が生み出された場合の帰属先<br>■ ⑫ 被験者を特定できないようにした上で，研究の成果が公表される可能性があること<br>■ ⑬ 研究に係る資金源，研究者等の企業・団体との関わり及び起こりうる利害の衝突<br>■ ⑭ 健康被害が生じた場合の補償について（研究に伴う補償がある場合にあっては，当該補償の内容を含む.）<br>■ ⑮ 問い合わせ，苦情等の窓口の連絡先等に関する情報<br>□ ⑯ 被験者からインフォームド・コンセントを受けることが困難な場合は，研究の重要性及び被験者の研究への参加が研究を実施するに当たり必要不可欠な理由（21＝②の場合）<br>□ ⑰ その他（　　　　　　　　　　）
24. インフォームド・コンセントを得ないで研究を行う場合<br><br>注：被験者または代諾者からのインフォームド・コンセントを得て研究を行う場合（21＝①または②または③）には本欄への記載は不要.	(1) 研究対象	□ ① 個人を特定できない人由来の材料及びデータのみを用いる（→ (3) 以下へ）<br>□ ② 個人を特定できる人由来の材料及びデータを用いる（→ (2) 以下へ）
	(2) (1) ＝②の場合，使用する材料及びデータの種類<br>注：(1) ＝①の場合は記載不要	□ ① 研究開始前に人体から採取された材料（試料）を用いる<br>（→24 (5) ②の措置を講じること）<br>□ ② 人体から採取された材料（試料）を用いず，既存のデータのみを用いる（→24 (5) ①の措置を講じること）<br>□ ③ 人体から採取された材料（試料）を用いず，新たに収集したデータを用いる（→24 (5) ②の措置を講じること）<br>□ ④ その他（　　　　　　　　　　）
	(3) 研究の性質	①研究が被験者に対する最小限の危険を超える危険を含むか<br>□ 含む<br>□ 含まない（その理由：　　　　　　）<br>②インフォームド・コンセントを免除することが被験者の不利益となるか<br>□ 不利益となる<br>□ 不利益とならない（その理由：　　　　　　）<br>③インフォームド・コンセントを免除しなければ，実際上，当該研究を実施できず，又は当該研究の価値を著しく損ねるか<br>□ 実施できる／研究の価値が損なわれない<br>□ 実施できない／研究の価値が損なわれる（その理由：　　　　）<br>④研究が社会的に重要性の高いものであると認められるか<br>□ 認められる（その理由：　　　　　　）<br>□ 認められない
	(4) インフォームド・コンセントに代わる措置	□ ① あり（→ (5) へ）<br>□ ② なし

10

	(5) (4) =①の場合，インフォームド・コンセントに代わる措置の内容	□ ① 研究対象者が含まれる集団に対し，資料の収集・利用の内容を，その方法も含めて広報する<br>□ ② 研究の実施についての情報を公開し，研究対象者となるものが研究対象者となることを拒否できるようにする<br>□ ③ できるだけ早い時期に，被験者に事後的説明（集団に対するものも可）を与える<br>□ ④ 長期間にわたって継続的に資料が収集又は利用される場合には，社会に，その実情を，資料の収集又は利用の方法も含めて広報し，社会へ周知される努力を払う<br>□ ⑤ その他（具体的に：　　　　　　　　　　　　　　　　　）
25. 既提供検体の利用	(1) 既提供検体の利用の有無	□ ① 利用する<br>■ ② 利用しない
	(2) 利用する（1=①）場合，同意を得ているか	□ ① 当該臨床研究への試料等の利用についての同意を得ており，当該同意に関する記録を作成する<br>□ ② 当該臨床研究への試料等の利用について同意を得ていない
	(3) 同意を得ていない（2=②）の場合，試料の匿名化の方法	□ ① 連結不可能匿名化<br>□ ② 連結可能匿名化であるが，対応表を保有していない<br>□ ③ 連結可能匿名化であり，対応表を保有している（→4 へ記載が必要）
	(4) 同意を得ないで，連結可能匿名化であり，対応表を保有している（3=③）場合	① 当該臨床研究への試料等の利用を明示せず，研究利用一般についての同意を得ているか<br>□ 同意を得ている（→②へ）<br>□ 同意を得ていない（→③～⑥へ記載が必要）
		② 試料提供時の同意が当該研究の目的と関連性があると<br>□ 合理的に認められる<br>*（研究の実施について試料等の利用目的を含む情報を公開する措置を講じること）*<br>□ 認められない（→③～⑥へ記載が必要）
		③ 研究の実施について利用目的を含む情報を<br>□ 公開する<br>□ 公開しない<br>④ 被験者が被験者となることを<br>□ 拒否できる<br>□ 拒否できない<br>⑤研究が公衆衛生の向上のために<br>□ 必要とされる（その理由：　　　　　　　　　）<br>□ 必要とされない<br>⑥被験者の同意を得ることが<br>□ 困難である（その理由：　　　　　　　　　　）<br>□ 可能である
26. 研究に係る資金源		□ ① 運営費交付金（教育研究費）<br>□ ② 省庁等の公的研究費（具体的に：科学研究費）<br>□ ③ 共同研究経費・受託研究経費<br>■ ④ 委任経理金（奨学寄附金）<br>□ ⑤ 研究費はとくに必要としない<br>■ ⑥ その他（具体的に：NPO 法人・熟年体育大学リサーチセンターから支給される）
27. 本研究と企業・団体との関わり		■ ① 本研究に企業等は関与しない<br>□ ② 共同研究として実施（相手先：　　　　　　　　　　　　）<br>［共同研究契約書の写しを添付のこと］<br>□ ③ 受託研究として実施（相手先：　　　　　　　　　　　　）<br>［受託研究契約書の写しを添付のこと］

11

	□ ④ 本研究に関与する企業等はあるが，共同研究・受託研究として実施しない（相手先：<br>関与の具体的内容：　　　　　　　　　　　　　　　）<br>（注：企業等へ研究成果などを提供する場合，個人情報保護・知的財産保護のため，共同研究・受託研究への移行が必要となる．<br>また，研究の性質によっては，補償等の関係から共同研究・受託研究への移行が必要となる．）
28. 起こりうる利害の衝突[ii]<br>（該当するものすべてにチェック）<br><br>注：本項目は，情報開示による利益相反への適切な対応を目的としており，該当した場合でも本学が許容できる範囲内であれば，特に研究計画の変更等は必要有りません．（研究推進・研究者保護が趣旨）．<br>本申請書末尾の解説を参照の上，記載すること．<br>なお，本項目に関する事項は必要に応じて「国立大学法人信州大学臨床研究に係る利益相反マネジメント規程」に従って審議されます．	□ ① 研究実施責任者及び研究者等が，本研究に関与する企業等から，本研究への関連の有無に関わらず共同研究費，受託研究費，委任経理金（奨学寄附金）等の研究資金を受け入れている．<br>□ ②本研究に関与する企業等との間に，本研究への関連の有無に関わらず機器や消耗品等の提供，依頼試験・分析，研究員の受入等がある．<br>（具体的に：　　　　　　　　　　　　　　　）<br>□ ③ 研究実施責任者及び研究者等が，本研究に関与する企業等との間に，役員・顧問等の非常勤を含む雇用関係，研究資金以外の謝金・報酬・給与の支払い等の経済的利益，知的財産権（特許や実用新案等）の共有・実施許諾・譲渡がある．または，当該企業の株式（未公開株・ストックオプションを含む）を所有している．<br>（個人情報を保護できる範囲で具体的に：　　　　　　）<br>□ ④ 研究実施責任者及び研究者等の，２親等以内の親族が，本研究に関与する企業等との間に，研究費の受入，報酬・給与等の経済的利益，経営関与，知的財産権（特許や実用新案等）の共有・実施許諾・譲渡がある．または，当該企業の株式（未公開株・ストックオプションを含む）を所有している．<br>（個人情報を保護できる範囲で具体的に：　　　　　　）<br>□ ⑤ 本研究の実施によって，本務である教育・研究・診療等に支障をきたす可能性がある．<br>（具体的に：　　　　　　　　　　　　　　　）<br>■ ⑥ 利害の衝突は起こり得ない
29. 研究に伴い被験者に健康被害等の有害事象が生じた場合の補償	□ ① 補償のための臨床研究保険を設定する［後日，保険の見積書を提出すること．］（項目4=①であり，6=①の場合は，この措置が必要）<br>□ ② 臨床研究保険以外の対処方法を講じる（具体的に：　　　）<br>□ ③ 本研究に関与する企業・団体等が補償する<br>（注：この場合，契約書により企業との間で合意を得ていることが必要）<br>■ ④ 保険診療による対応<br>□ ⑤ 有害事象は生じ得ないため，補償はない（有害事象が生じ得ない理由を記載：　　　）<br>□ ⑥ その他（具体的に：　　　）
30. 研究実施にあたっての確認事項<br>（該当するものにすべてチェックを入れること）	【共通事項】<br>■ ① 研究実施責任者に，研究を適正に実行するために必要な専門的知識及び臨床経験が十分にあること．<br>■ ② 研究実施責任者は，臨床研究に関する重篤な有害事象及び不具合等の発生を知ったときは，直ちにその旨を臨床機関の長に通知しなければならない。<br>■ ③ 研究実施責任者は，研究により期待される利益よりも起こりうる危険が高いと判断される場合又は研究により十分な成果が得られた場合には，当該研究を中止し，又は終了すること．また，遅延なく，その旨を研究機関の長へ報告すること．<br>■ ④ 研究実施責任者は，研究を終了するまでの間，研究に関する国内外における学会発表，論文発表等の情報（以下「発表情報等」という．）について把握しておくとともに，把握した当該発表情報等につい

	て，研究機関の長に対し，報告すること．<br>■ ⑤ 研究実施責任者は，毎年一回，研究の進捗状況，または研究が終了したときには，その旨及び結果の概要を文書により研究機関の長へ報告すること．<br>■ ⑥ 研究実施責任者は，研究を実施するに当たり，被験者の個人情報の保護のために必要な措置を講じていること．<br>■ ⑦ 研究実施責任者は，研究終了後においても，被験者が当該研究の結果により得られた最善の予防，診断及び治療を受けることができるよう努めること．<br>■ ⑧ 研究者等は，臨床研究の実施に先立ち，臨床研究に関するその他臨床研究の実施に必要な知識についての講習その他必要な教育を受けなければならない．<br>■ ⑨ 研究者等は，研究を実施するに当たっては，一般的に受け入れられた科学的原則に従い，科学的文献その他科学に関連する情報源及び十分な実験に基づいていること．<br>■ ⑩ 研究者等は，研究の結果を公表する場合には，被験者を特定できないように行うこと．<br>【健康に影響を与えるような行為を伴う人を対象とする研究（いわゆる介入研究）の場合】<br>■ ⑪ 臨床経験が十分にある医師による適切な助言を得ていること．（研究チームに医師が含まれない場合は医師名を記載：　　　　　）<br>【他の研究機関と共同で研究を実施する場合】<br>□ ⑫ 研究実施責任者は，当該他の研究機関の研究実施責任者に対し，研究に起因する重篤な有害事象を報告すること．<br>□ ⑬ 研究実施責任者は，当該他の研究機関の研究実施責任者に対し，把握した発表情報等について報告すること．<br>【環境に影響を及ぼすおそれのある研究を実施する場合又は研究の実施に当たり動物を使用する場合】<br>□ ⑭ 研究者等は，環境への影響や動物の使用に対して十分な配慮をしていること．<br>□ ⑮ その他（　　　　　　　　　　　　　　　　）
31. その他記載すべき事項	

---

[i] 「臨床研究に関する倫理指針」における個人情報の定義：生存する個人に関する情報であって，当該情報に含まれる氏名，生年月日その他の記述等により特定の個人を識別することができるもの（他の情報と容易に照合することができ，それにより特定の個人を識別することができることとなるものを含む．）をいう．なお，死者に係る情報が同時に，遺族等の生存する個人に関する情報である場合には，当該生存する個人の個人情報となる．代表的な個人情報には，氏名，生年月日，住所，電話番号のほか，患者ごとに記録された診療録番号等の符号を含む情報等が考えられるが，この指針における個人情報となるか否かは具体的な状況に応じて個別に判断することとなる．

[ii] 「利害の衝突」とは，研究者等が研究の実施や報告の際に，金銭的な利益やそれ以外の個人的な利益のためにその専門的な判断を曲げてしまう（もしくは曲げたと判断される）ような状況や，当該研究の実施によって本務である教育・研究・診療等に支障をきたすような状況を示す．（前者を狭義の「利益相反」，後者を「責務相反」という．）

この狭義の「利益相反」は，研究者やその親族等が資金提供や研究依頼のあった者・団体（政府，財団，企業等）から臨床研究に係る資金源の他に機器や消耗品等の提供を受けること，実施料を受け取ること，その株式を所有（未公開株やストックオプションを含む）すること，特許権を共有・譲渡されること，講演料や著述料の支払いを受けていること等である．

それ以外の利害の衝突とは，研究者やその親族等が資金提供や研究依頼のあった者・団体との間に顧問等の非常勤を含む雇用関係があることや，親族や師弟関係等の個人的関係があることなど，研究者等の関連組織との関わりについての問題などが考えられる．

上記の様な利害の衝突の中でも，明示的に確認することができる①資金源等の金銭上の利害の衝突②研究者等の関連組織との関わりについては少なくとも記載するべきである．
