# Supplementary material for: Effects of milk product intake on thigh muscle strength and NFKB gene methylation during home-based interval walking training in older women: A randomized, controlled pilot study
Source: PLoS One. 2017 May 17;12(5):e0176757. doi: 10.1371/journal.pone.0176757 (PMC5435182; doi:10.1371/journal.pone.0176757)
Supplement: S3 Protocol — (DOCX) [file pone.0176757.s004.docx]

Shinshu University School of Medicine Human Genetic Study Application Form ver. 090401

August 24, 2011

| 1. Classification I  (Select only one. Another number should be separately applied.) | | [3] Human genetic study | | | |
| --- | --- | --- | --- | --- | --- |
| 2. Classification II | | [1] Interventional study (→it must be applied to the Ethics Review Board as a clinical research plan.) | | | |
| 3. Name of plan | | Verification of the preventive effects of the combination of exercise and supplement consumption on lifestyle-related diseases---Investigation of blood cell DNA methylation and its verification from the viewpoint of epigenetics | | | |
| 4 Chief investigator  (Affiliation/position) | | Name:  Shun'ichiro Taniguchi | Affiliation:  Shinshu University Graduate School of Medicine | Position:  Professor | Date of educational or audiovisual training (August 19, 2011) |
| 5 Persons in charge of the study (Affiliation/position), telephone/fax/e-mail | | Name:  Shun'ichiro Taniguchi | Affiliation:  Shinshu University Graduate School of Medicine | Position:  Professor | Date of educational or audiovisual training (August 19, 2011) |
|  |  | TEL/FAX  37-2679/37-2724 |  | e-mail:  stangch@shinshu-u.ac.jp |  |
| 6. Other persons in charge of the study  (Affiliation/position), role | | Name:  Hiroshi Nose  Shizue Masuki | Affiliation:  Shinshu University Graduate School of Medicine | Managerial position:  Professor  Assistant professor | Date of educational or audiovisual training (February 18, 2009) |
|  |  | Sohichiro Nakamura  Shigeru Katayama | Faculty of Agriculture | Professor  Assistant professor | Date of educational or audiovisual training (February 18, 2009 |
|  |  | Hiroshi Fujii |  | Professor | (August 19, 2011) |
|  |  | Shigeaki Hida  Shigenari Hashimoto | Shinshu University Graduate School of Medicine | Associate professor  Assistant professor | Date of educational or audiovisual training  (August 24, 2011)  (May 7, 2009) |
| 7. Person managing personal information in this analysis (Affiliation/position) | | Jinko Sawashita, Assistant professor, Department of Aging Biology | | | |
| 8. Type of analysis | | [1] Analysis involving a single group | | | |
| 9 Disease to be analyzed | | Pre-lifestyle-related-disease state | | | |
| 10. Disclosure of the results of inspection by the Ethics Review Board | | [1] The above information may be disclosed. | | | |
| 11. Genes to be analyzed  (Individual genes should be reviewed, and their relationships with diseases to be analyzed should be described. When the number of genes is large, only a representative case should be presented. Although an appendix may be attached, a clinical diagnosis and study of which the clinical significance has been established should not be included together.) | | As genes involved in elderly persons’ responsiveness to exercise prescriptions or lifestyle-related diseases (candidate genes to be analyzed), genes involved in chronic inflammation will be analyzed:  [1] NF-KB, ASC, cytokines, or their receptors involved in inflammation  [2] Insulin, oncogenes, or tumor suppressor genes involved in diabetes or cancer. | | | |
| 12. Subjects to be analyzed  (In the parenthesis, it should be described that criteria/strategies/concepts for selecting persons who provide genes are rationally adopted. if diseases or abnormalities in drug responsiveness are present, a method to disclose the disease name and its findings should be described.) | | [5] Others (Persons who had attended Jukunen Taiikudaigaku project, and received dietary intervention and exercise prescriptions, consenting to DNA collection) | | | |
| 13. Purpose/necessity/significance of analysis | | [1] Purpose of this study: Exercise prescriptions for the elderly have been conducted at Matsumoto City Jukunen Taiikudaigaku since 1997, contributing to health promotion and a reduction in health expenditure. As there were individual differences in the effects of exercise prescriptions, the necessity of genetic analysis was suggested. A DNA bank was established with a subsidy for genetic background-based individualized exercise guidance research in collaborative business by the Ministry of Education, Culture, Sports, Science and Technology in 2006 to 2010. The purpose of this study is to conduct epigenetic analysis of improvements in symptoms of lifestyle-related diseases by combining exercise prescriptions with dietary intervention.  [2] Necessity/significance: In addition to accumulated genetic SNP data, we recently clarified that inflammation-associated gene methylation depended on exercise prescriptions. In this study, we will investigate the state of lifestyle-related disease-associated gene methylation by combining exercise prescriptions with dietary intervention. Such an epigenetic study has the essential of preventive medicine; it is significant. | | | |
| 14. Methods  * If the detection sensitivity can be expected, it should be described. | | 1) Data collection: For subjects in whom conventional exercise prescriptions alone do not reduce symptoms of lifestyle-related diseases, such as hyperglycemia, dietary intervention will be performed, and the association between its effects and the epigenetic effects of symptom- and lifestyle-related-disease-associated genes will be analyzed. For this purpose, an explanatory meeting will be held, and informed consent regarding participation in this study will be obtained after a specific period. In persons consenting to participation, 14 ml of blood will be collected for DNA extraction in the Department of Clinical Laboratory, Shinshu University Hospital, and the collaborative institution (Jukunen Taiikudaigaku). Blood samples will be sent to our university, and anonymized at the time of blood collection. Other clinical data on the intensity of exercise, dietary intake in the presence of intervention, physical strength, health, diet, and health expenditure will be collected, accumulated on an e-health promotion system, and accumulated/strictly managed according to the Personal Information Protection Law.  2) Analysis of genetic methylation: Personal methylation at 10% of the maximum and minimum dietary intervention and exercise prescription effects before and after intervention will be compared. Inflammation-associated genes showing preliminary exercise responsiveness will be primarily analyzed. Quantitative methylation measurement will be conducted using a device to be introduced in the Instrument Analysis Division of the Human Environmental Science Research Support Center. | | | |
| 15. Period  * Five years at maximum | | Start: from September 6, 2011  Completion: until August 31, 2014 | | | |
| 16. Expected results  (In the case of clinical gene diagnosis, its usefulness, sensitivity, and specificity, as well as the accuracy of positive/negative results, should be described, and the literature should be attached.) | | The lifestyle-related disease-preventing methods obtained as the study outcome will be introduced as new guidance methods in Jukunen Taiikudaigaku. Therefore, both experimental and control groups may be guided based on the latest study outcome in the future, and conventional health promotion effects may inflammation-associated gene modification matched to an improvement in inflammation may reduce inflammation. | | | |
| 17. Expected risks/disadvantages | | There may be no risks/disadvantages based on the courses of exercise prescriptions and gene analysis. | | | |
| 18. Type and volume of samples, collection methods | | Blood, approximately 14 mL (approximately 7 mL before prescription, 7 mL after prescription, total: 14 mL) | | | |
| 19. Number of persons to be analyzed | | 180 | | | |
| 20. Protection of personal information | (1) Methods |  | | | |
|  |  | [2] Linkable anonymization (it is necessary to describe (2))  Reason:  Clinical data obtained after sample collection (including information on the prognosis) are necessary for final analysis. | | | |
|  |  |  | | | |
|  | (2) Linkable anonymization ((1) = [2]): Correspondence table-managing method | [1] Computers separated from other computers must be used. The data should be recorded in an external memory medium, key-locked, and strictly stored. | | | |
| 21. Addition/change of the protocol | | [1] Expected (Outline: the same population will be added.) | | | |

II Collaborative institution

| 1. Name of a collaborative institution | [1] It can be identified.  (Institution/investigator: Sohichiro Nakamura, Faculty of Agriculture, Shinshu University) |
| --- | --- |
| 2. If the name of a collaborative institution cannot be identified (1 = [2], [3]), which institution will participate in the future? |  |
| 3. Role of our hospital in the collaborative study | [1] Sample collection  [2] Gene analysis  [3] Data collection/analysis |
| 4. Role of other institutions in the collaborative study | [4] Others (Preparation of adjuvant foods) |
| 5. Presence or absence of explanatory and consent documents prepared by other institutions | [2] No documents |
| 6. If samples are collected in other institutions, are they acceptable? | [2] Not acceptable. |
| 7. If samples are provided by other institutions (6 = [1]), will they be anonymized? | Not applicable. |
| 8. Will samples collected in our hospital be submitted to another institution? | [2] They will not be submitted. |
| 9. If samples are submitted to another institution (8 = [1]), will they be anonymized? | Not applicable. |

III Informed consent

| 1. Person who explains the purpose/contents of genetic analysis to subjects (Affiliation/position) | Shun'ichiro Taniguchi (Professor, Graduate School of Medicine, Shinshu University) |
| --- | --- |
| 2. Explanatory and consent forms for genetic analysis subjects | [1] Attached. |
| 3. Matters to be described in the explanatory document | [1] The protocol was examined at the “Genetic Analysis Ethics Review Board”, and approved by the Director of the School of Medicine, Shinshu University.  [2] Explanations regarding genetics/genes/genetic analysis  [3] Concrete procedures (informed consent/storage of a consent form)  [4]-l Genetic diagnosis and study cooperation are voluntary.  [4]-2 Subjects can withdraw from this study at any time in writing.  [4]-3 There may be no disadvantage even if subjects do not consent to participation or withdraw from this study.  [4]-4 Discarding of samples/study results related to withdrawal  [5] Reasons why subjects were selected as donors (genetic diagnosis or study cooperation was proposed)  [6] Name/position of the chief investigator  [7] Significance/purpose/necessity of analysis  [8] Analysis methods/period (expected addition/change of the study contents)  [10] Disclosure of the protocol  [11] Advantages/disadvantages for genetic diagnosis subjects or sample donors, expected results  [12] Protection of personal information  [14] Disclosure of the results of genetic analysis  [15] Publication of the study outcome with anonymization  [16] Storage/use/discarding of samples and medical information during the analysis period and after its completion (including their utilization in other studies and expected study contents)  [17] Information on the utilization of genetic counseling  [18]-1 Expenditure  [18]-2 Matters regarding the value of sample provision  [18]-3 Analysis-related intellectual property right and its assignment  [18]-4 Preparation of analytical funds  [19]-l Inquiry to  [19]-2 Complaint counter |

IV Legal representative

| l. Will the following samples be analyzed? | [6] Cases [1] - [5] will not be included (it is unnecessary to describe Item 2 or below.) |
| --- | --- |
| 2. If corresponding to IV. 1 [1] - [4], direct advantages related to this analysis | Not applicable. |
| 3. If there is no advantage (2 = [2] or 1 = [4]), reasons why such samples must be provided for treatment/research and importance of diagnosis/research | Not applicable. |
| 4. If corresponding to IV. 1 [1] - [4], basic concept regarding the selection of a legal representative | Not applicable. |
| 5. If samples are derived from dead persons (1 = [5]), basic concept regarding the selection of bereaved families | Not applicable. |

V Disclosure of genetic information (Will genetic information be disclosed to the subject or legal representative?)

| l. Presence or absence of the disclosure of genetic information obtained from genetic analysis to the subject or legal representative |  |
| --- | --- |
|  | [2] Not disclosed as a rule.  Reason:  [1] Currently, such information is not significant for the assessment/management of individuals’ health status.  (Concretely: ) |

VI Utilization of provided samples

| 1. Will such samples be used? | [2] No use. |
| --- | --- |
| 2. Presence or absence of a consent regarding genetic analysis if the sample is utilized (1 = [1]) | Not applicable. |
| 3. In the absence of a consent (2 = [2], [3]), will a consent be newly obtained? | Not applicable. |
| 4. Sample-anonymizing method in the absence of a consent (3 = [2]) | Not applicable. |
| 5. Linkable anonymization in the absence of informed consent (4 = [2]) | Not applicable. |
| 6. Timing of provided samples’ arrival | Not applicable. |

VII Provision of samples/genetic information to public research organizations other than collaborative institutions (Consignment of samples to a cell/gene/tissue bank: it is unnecessary to describe this column. Please describe in IX-3.)

| 1. Will samples or genetic information obtained from them be provided to other public research organizations? | [2] Not provided. |
| --- | --- |
| 2. Organization (1 = [1]) | Not applicable. |
| 3. Presence or absence of anonymization at the provider if samples or genetic information is provided (1 = [1]) | Not applicable. |
| 4. Presence or absence of genetic analysis at the organization if samples or genetic information is provided (1 = [1]) | Not applicable. |
| 5. If samples or genetic information is provided (1 = [1]), will they be repeatedly or continuously provided? | Not applicable. |

VIII Provision to profit organizations (including cases in which analysis is committed to a third organization)

| 1. Will samples or genetic information obtained from them be provided to other profit organizations or private institutions? | [2] Not provided. |
| --- | --- |
| 2. Institution | Not applicable. |
| 3. Anonymization method at the provider | Not applicable. |
| 4. Director of the institution, responsibility-taking system, and contents of the expected contract | Not applicable. |

IX Storage of samples

| 1. Storage of samples during the study period | (1) Presence or absence of the storage of samples during the study period | [1] Stored in the institution (→ it is necessary to describe the following columns.) |
| --- | --- | --- |
|  | (2) Storage method and period ((1) = [1] or [2]) | [2] Nucleic acid is extracted/stored (Storage period: until date/year) |
|  | (3) Necessity of storage ((1) = [1] or [2]) | [1] For additional examination to assure the accuracy of the results of genetic analysis |
|  | (4) Person responsible for the storage of samples | [2] Managed by investigators other than [1] (→ The name of the person responsible for the manage of samples should be described.)  Affiliation: Department of Aging Biology  Position: Assistant professor  Name: Jinko Sawashita |
| 2. Storage of samples after the study period | (1) Presence or absence of the storage of samples after the completion of this study | [1] Stored in the institution (Place/School of Medicine, 2011 to 2016) |
|  | (2) Storage method and period ((1) = [1] or [2]) | [2] Nucleic acid is extracted/stored. |
|  | (3) Necessity of storage/reasons for storage ((1) = [1] or [2]) | [1] To utilize samples/materials for future studies  (expected study contents: the same as this time ) |
|  | (4) Type of anonymization ((1) = [1] or [2]) | [2] Linkable anonymization |
| 3. Consignment of samples to a cell/gene/tissue bank | (1) Schedule | [2] No schedule. |
|  | (2) Organization for consignment ((1) = [1]) | Not applicable. |
|  | (3) Anonymization method ((1) = [1]) | Not applicable. |
| 4. Sample-discarding method | | [2] Burning with anonymization |

X Genetic counseling

| l. Necessity of genetic counseling | [2] Necessary in some cases. |
| --- | --- |
| 2. (1 = [1] or [2]): Institution and person responsible for genetic counseling (qualification, previous educational training) | [1] Yoshimitsu Fukushima (Specialist in clinical genetics) et al., Department of Genetic Diagnosis and Treatment, Shinshu University Hospital |

XI Source of funds regarding this study

| Source of funds regarding this study | [1] Subsidy for operating costs (education/research funds)  [2] Ministries’ and agencies’ public grants (special expenditures from the Ministry of Education, Culture, Sports, Science and Technology in 2011) |
| --- | --- |

XII Relationship of this study with companies/corporations

| Relationship of this study with companies/corporations | [1] No company/corporation will be involved in this study. |
| --- | --- |

XIII Conflict of interest

| 28. Conflict of interest^i^ (Please check all corresponding items.)  Note: The purpose of this item is to adequately manage information disclosure-related conflict of interest. Even if the situation corresponds to this, there may be no need to change the study plan when it is within the permissible range of our university (for study promotion/protection of investigators).  Please describe this in reference to explanations at the end of this application form.  However, matters regarding this item will be examined according to the “Shinshu University Regulations for Conflict-of-Interest Management regarding Clinical Research, if necessary. | [6] There may be no conflict of interest. |
| --- | --- |

XIV Matters to be mentioned

| 1. Presence or absence of matters to be mentioned | [2] The rapidity of inspection is required (Reason: Analysis will be started in early September). |
| --- | --- |

XV Disclosure of the protocol

| 1.Disclosure of this protocol for genetic analysis | [1] Possible. |
| --- | --- |
| 2. A portion is impossible to disclose (1 = [2]): Item numbers to be closed | Not applicable. |
| 3. Disclosure (portion) is impossible (1 = [2] or [3]): Reasons | Not applicable. |

XVI Others

| Free description (wishes for inspection) | No description. |
| --- | --- |
