# Supplementary material for: Effects of milk product intake on thigh muscle strength and NFKB gene methylation during home-based interval walking training in older women: A randomized, controlled pilot study
Source: PLoS One. 2017 May 17;12(5):e0176757. doi: 10.1371/journal.pone.0176757 (PMC5435182; doi:10.1371/journal.pone.0176757)
Supplement: S4 Protocol — (PDF) [file pone.0176757.s005.pdf]

遺伝子解析計画名：運動と補助食品摂取の併用による生活習慣病予防効果の検証一血液細胞のＤＮＡメチル化を調べそのエピジェネティックスの側面からの検証

（信大医遺伝子解析様式１）

信州大学医学部「ヒト遺伝子解析」計画書　ver. 090401

提出日	平成23年8月24日
提出番号＊	
諾否決定日＊	
諾否＊	
承認番号＊	

＊ 申請者においては記入しないこと

以下，項目番号を変更しないこと。

I. 概要

1. 分類I（1 個のみ選択。別番号は別申請すること）	□ ①「臨床的遺伝子診断」<br>□ ②「遺伝子診断研究」<br>■ ③「ヒト遺伝子研究」<br>□ ④「体細胞遺伝子解析」			
2. 分類II	■ ①介入を伴う研究（→臨床研究計画として医倫理委員会にも申請が必要）<br>□ ②非介入研究			
3. 計画名	運動と補助食品摂取の併用による生活習慣病予防効果の検証一血液細胞のＤＮＡメチル化を調べそのエピジェネティックスの側面からの検証			
4. 実施責任者氏名（所属・職名）	氏名<br>谷口俊一郎	所属<br>医学系研究科	職名　教授	■教育研修受講日または視聴日<br>（2011年8月19日）
5. 実施担当者氏名（所属・職名），電話・ファックス・電子メール	氏名<br>谷口俊一郎	所属<br>医学系研究科	職名　教授	■教育研修受講日または視聴日<br>（2011年8月19日）
	TEL／FAX<br>37-2679/37-2724		e-mail:stangch@shinshu-u.ac.jp	
6.その他の実施担当者の氏名（所属・職名），役割	氏名（役割）<br>能勢　博<br>増木静江<br>（運動処方）	所属<br>医学系研究科	職名<br>教授<br>助教	■教育研修受講日または視聴日<br>（2009年2月18日）
	中村宗一郎<br>片山　茂<br>（補助食処方）<br>藤井　博<br>（成分解析）	農学部	教授<br>助教<br>教授	■教育研修受講日または視聴日<br>（2009年2月18日）<br>（2011年8月19日）
	肥田重明<br><br>橋本繁成<br>（遺伝子解析）	医学系研究科	准教授<br><br>助教	■教育研修受講日または視聴日<br>（2011年8月24日）<br>（2009年5月7日）
7.本解析における個人情報管	加齢生物学分野助手・澤下仁子			

遺伝子解析計画名：運動と補助食品摂取の併用による生活習慣病予防効果の検証ー血液細胞のＤＮＡメチル化を調べそのエピジェネティックスの側面からの検証

理者氏名（所属・職名）	
8. 解析の形態	■ ① 単独グループによる実施<br>□ ② 多施設共同研究の主任研究者（→Ⅱへの記載が必要）<br>□ ③ 多施設共同研究への参加（→Ⅱへの記載が必要）<br>（主任施設名：　　　　）<br>（他施設の倫理委員会による承認証明書の添付：□あり □なし）<br>□ ④その他（　　　　）
9. 対象とする疾患名	前生活習慣病状態
10. 倫理委員会審査結果の公開	※下記の項目を委員会のホームページで公開します.<br>1. 研究計画名<br>2. 研究者氏名（申請者・委員会当日の説明者）<br>3. 審査結果<br>■ ① 上記の情報のまま公開可<br>□ ② 上記の情報のままでは公開不可のため，以下のように訂正を求める<br>（訂正内容を具体的に記載：　　　　）
11. 対象とする遺伝子名<br>（個々の遺伝子について概説し，対象疾患との関係がどの程度明らかになっているかを記載。遺伝子数が膨大な時は代表例のみあげ，別添でもよいが，臨床的意義が確立している臨床診断と研究とが一緒に含まれないよう注意すること。）	高齢者の運動処方への反応性や生活習慣病に関与すると考えられる遺伝子、解析候補遺伝子としては、特にその誘引と考えられる慢性炎症状態に関わる遺伝子群とする。<br>① 炎症に関与するNF-κBやASC、サイトカインまたそれらの受容体<br>② 糖尿病、がんなどに関与するインスリン、癌遺伝子、癌抑制遺伝子など<br>を中心として解析を行う。
12. 解析対象者<br>（提供者を選ぶ基準・方針・考え方を，合理的に選択していることがわかるように括弧内に記載。疾患や薬剤反応性異常を有する場合等は，病名や病態像の開示方法も記載のこと）	□ ①診断が確定している患者（　　　　）<br>□ ②罹患が疑われる患者（　　　　）<br>□ ③親族（　　　　）<br>□ ④コントロール（　　　　）<br>■ ⑤その他（熟年体育大学受講者で食介入と運動処方を行い、ＤＮＡ採取に同意をして頂いた方々）
13. 解析の目的・必要性・意義	**① 研究目的**：平成9年より「松本市熟年体育大学」で高齢者への運動処方を実施し、健康増進、医療費削減に効果を挙げている。運動処方効果に個体差が存在することから、遺伝子解析の必要性が示唆され、平成18-22年度文科省連携融合事業で遺伝背景を考慮した個別運動指導研究に対して補助を受け、DNAバンクを構築した。今回は、運動処方に食の介入を加え生活習慣病の各症状改善についてエピジェネティックな解析を行うことを目的とする。<br>**② 必要性・意義**：蓄積された遺伝子ＳＮＰデータに加え、最近、炎症関連遺伝子メチル化が運動処方によって変化することを明らかにした。今回は、運動処方に食の介入効果を加え、生活習慣病に関連する遺伝子のメチル化の状態を調べる。このようなエピジェネティックス研究は、予防医学の本質に迫るもので、その意義は大きい。
14. 方法<br>＊ 検出感度が予想できる場合は記載する。	**１）データの収集**：従来の運動処方だけでは高血糖などの生活習慣病の症状が改善しない被験者に食の介入を行い、その効果について同症状と生活習慣病関連遺伝子のエピジェネティック効果との関連を解析する。そのために、説明会を行い、一定期間を置いて研究への参加承諾を得る。承諾者に対して、本学附属病院臨床検査科及び共同研究機関（熟年体育大学）でDNA抽出用に14 ml の血液を採取する。採取した血液は本学

	へ送付、採決時に匿名化する。その他の臨床データである運動量、介入した食の摂取状況、体力、健康データ、食生活、医療費についてデータを収集、e-ヘルスプロモーションシステムに蓄積し、個人情報保護法に則り、厳重に管理する。<br>**２）遺伝子メチル化解析**：食介入と運動処方効果の最大と最少それぞれ約 10%の個人のメチル化を介入前後で比較検討し解析する。解析する遺伝子は、予備的に運動反応性がみられた炎症関連遺伝子を中心として行う。定量的メチル化測定はヒト環境科学研究支援センター機器分析部門に導入される予定の装置を用いて行う。
15. 期間<br>＊最大５年	開始：☐ ①承認日より<br>☐ ② 23年９月６日より<br>終了： 平成26年８月31日まで
16. 予測される成果<br>（臨床的遺伝子診断の場合は，遺伝子診断の妥当性・有用性，感度，特異性，陽性・陰性結果の正診率などについても記載し，文献も添付することが望ましい）	研究成果として明らかになった生活習慣病予防方法は、熟年体育大学の新たな指導~~方法として導入する~~。したがって、実験群とコントロール群ともに、今後、最新の研究成果に基づき指導を受けることができ、従来とおりの健康増進効果が得られる。炎症状態の改善に対応する炎症関連遺伝子修飾状態は炎症を抑える方向に動くと予想される。
17. 予測される危険・不利益	現在までの運動処方、遺伝子解析の経過からすると特に考えられない。
18. 検体の種類と量，その採取方法	血液約 14ｍＬ（処方前約 7ｍＬ、処方後 7ｍｌで合計 14ｍＬ）
19. 解析対象予定人数	180名
20. 個人情報の保護　(1) 方法	☐ ①連結不可能匿名化
	■ ②連結可能匿名化（(2) への記載が必要）<br>理由：<br>☐解析結果を検体提供者の診療に反映させるため。<br>■検体採取後に明らかになった臨床データ（予後の情報など）も最終的な解析に必要であるから。<br>☐その他（ ）
	☐ ③その他の方法（具体的に： ）<br>匿名化しない理由（ ）
(2) 連結可能匿名化（(1) =②）の場合，対応表の管理方法	■ ①他のコンピュータと切り離されたコンピュータを使用し，外部記憶装置に記録させ，そのメディアは，鍵をかけて厳重に保管。<br>☐ ②筆記による原簿として鍵をかけて厳重に保管。<br>☐ ③その他（ ）
21. 解析計画の追加・変更	■ ①予想される（概要：内容は同じ母集団の追加のため）<br>☐ ②予想されない

II. 共同研究機関

1. 共同研究機関名	■①特定できる<br>（研究機関名・研究者氏名：信州大農学部中村宗一郎）<br>☐ ②特定できない（理由： ）（2への記載が必要）<br>☐ ③一部特定できない（理由： ）（2への記載が必要）

遺伝子解析計画名：運動と補助食品摂取の併用による生活習慣病予防効果の検証ー血液細胞のＤＮＡメチル化を調べそのエピジェネティックスの側面からの検証

6

2.共同研究機関名を特定できない（1=②，③）場合，将来参加が予測される共同研究機関はどのような施設か？	
3.共同研究における当院の役割	■ ①検体の採取<br>■ ②遺伝子の解析<br>■ ③データの収集と解析<br>□ ④その他（ ）
4.共同研究における他施設の役割	□ ①検体の採取<br>□ ②遺伝子の解析<br>□ ③データの収集と解析<br>■ ④その他（補助食の準備 ）
5.他施設作製の説明文書および同意文書の有無	□ ①あり（別添）<br>■②なし
6.他施設で検体を採取する場合，採取した検体の提供を受けるか？	□ ①提供を受ける（7への記載が必要）<br>■ ②提供を受けない
7.他施設から検体の提供を受ける（6=①）場合，匿名化を行うか？	□ ①検体を採取した共同研究機関で行う（原則）<br>□ ②当院で行う（理由： ）<br>□ ③行わない（理由： ）
8.当院で採取した検体を他施設に提供するか？	□ ①提供する（9への記載が必要）<br>■ ②提供しない
9. 他施設に検体を提供する（8=①）場合，匿名化を行うか？	□ ①当院で行う（原則）<br>□ ②提供先の共同研究機関で行う（理由： ）<br>□ ③行わない（理由： ）

III. インフォームド・コンセント

1.遺伝子解析対象者等に説明を行う者の氏名（所属・職名）	谷口　俊一郎（医学系研究科・教授）
2.遺伝子解析対象者等に渡す説明文書，同意文書	■ ①別添<br>□ ②（中心となる）他施設作製の説明文書・同意書を別添
3. 説明文書の記載事項	■①当該解析計画は「遺伝子解析倫理委員会」で審査され，信州大学医学部長によって承認されたものであること<br>■②遺伝・遺伝子・遺伝子解析などについての説明<br>■③具体的な手順（説明と同意・同意書の保管等）<br>■④-1　遺伝子診断を受けることおよび研究協力は任意であること<br>■④-2 いつでも文書により同意が撤回できること<br>■④-3 同意しないことや同意の撤回により不利益な対応を受けないこと<br>■④-4 同意撤回による検体・研究結果等の廃棄について<br>■⑤提供者として選ばれた（遺伝子診断，あるいは研究協力を提案された）理由<br>■⑥実施責任者の氏名・職名<br>■⑦解析の意義・目的・必要性<br>■⑧解析の方法・期間（将来研究内容の追加・変更が予想される場合はその旨）<br>□ ⑨代諾者によるインフォーム・ドコンセントが必要な場合は検体が必要な理由・解析の重要性

遺伝子解析計画名：運動と補助食品摂取の併用による生活習慣病予防効果の検証一血液細胞のＤＮＡメチル化を調べそのエピジェネティックスの側面からの検証

7

	■⑩実施計画書等の開示について<br>■⑪遺伝子診断の受検者あるいは検体提供者にもたらされる利益および不利益・予測される結果<br>■⑫個人情報の保護の方法<br>□⑬-1 検体や個人情報を他の機関に提供する可能性，その方法<br>□⑬-2　バンクに検体を提供する場合は，バンクの学術的意義，匿名化の方法，運営機関の名称，責任者の氏名<br>■⑭遺伝子解析結果の開示について<br>■⑮研究成果が，匿名化の上公表されうること<br>■⑯解析期間中及び終了後の検体，診療情報等の保存・使用・廃棄の方針（他の研究への利用の可能性とその予測される研究内容を含む）<br>■⑰遺伝カウンセリングの利用に係る情報<br>■⑱-1 費用負担<br>■⑱-2 検体提供の対価に関する事項<br>■⑱-3 解析から知的財産権が生じうることとその帰属先<br>■⑱-4 解析資金の調達方法<br>■⑲-1 解析に関する間合せ先<br>■⑲-2 苦情の受付先<br>□　⑳その他（　　　　　　　　　　　　）

IV. 代諾

1. 次に該当するものからの検体を解析の対象とするか？	□　①16 歳以上の未成年者からの検体<br>□　②16 歳未満の者からの検体<br>□　③判断能力が不十分なため有効なインフォームド・コンセントをなしえない成年者からの検体<br>□　④正確な診断を開示していない患者からの検体<br>□　⑤死者からの検体（生前における明示的な意思に反していない場合に限る）<br>■　⑥ ①一⑤は対象としない（2 以下への記載は不要）
2. 1 で①一④のいずれかに該当する場合，本解析によって本人にもたらされる直接の利益	□　①あり（理由：　　　　　）<br>□　②なし（3 への記載が必要）
3. 本人に利益がもたらされない（2=②あるいは 1=⑤）場合，その検体提供を受けなければ診療・研究が成りたたない理由及び診断・研究の重要性	
4. 1 で①一④のいずれかに該当する場合，代諾者選定に関する基本的な考え方	□　①任意後見人，親権者，後見人や保佐人が定まっているときはその人。いないときは，提供者本人の配偶者，成人の子，父母，成人の兄弟姉妹もしくは孫，祖父母，同居の親族又はそれらの近親者に準ずる人の中から，関係者間で協議して選定してもらう<br>□　②その他（　　　　　　　　　　　）
5. 死者からの検体（1=⑤）の場合，遺族の選定に関する基本的な考え方	□　①提供者本人の配偶者，成人の子，父母，成人の兄弟姉妹もしくは孫，祖父母，同居の親族又はそれらの近親者に準ずる人の中から，関係者間で協議して選定してもらう<br>□　②その他（　　　　　　　　　　　）

遺伝子解析計画名：運動と補助食品摂取の併用による生活習慣病予防効果の検証ー血液細胞のＤＮＡメチル化を調べそのエピジェネティックスの側面からの検証

8

V. 遺伝情報の開示（解析された遺伝情報を本人あるいは代諾者に伝えるか）

1. 遺伝子解析の結果得られた遺伝情報を本人あるいは代諾者に開示するか？	□ ①希望に応じて開示する<br>理由：<br>□①本人の健康管理に役立つと考えられるから<br>□②その他（ ）<br>■②原則として開示しない<br>理由：<br>■①現時点では，当該情報が個人の健康状態の評価や管理に十分な意義がないから<br>（具体的に： ）<br>□②連結不可能匿名化を行うから<br>□③その他（ ）

VI. 既提供検体

1. 既提供検体を用いるか？	□ ①用いる（2以下への記載が必要）<br>■ ②用いない
2. 用いる（1=①）場合，その検体は遺伝子解析を行う同意を得ているか？	□ ①遺伝子解析への利用について同意を得ている（同意書を必ず添付する）<br>□ ②遺伝子解析への利用を明示せずに研究利用一般についての同意を得ている<br>□ ③研究利用についての同意を得ていない
3. 得ていない（2=②，③）場合，新たに同意をえるか？	□ ①新たに同意を得る（原則）<br>□ ②新たに同意を得ない（4以下への記載が必要）
4. 同意を得ない（3=②）場合，検体の匿名化は？	□ ①連結不可能匿名化する<br>□ ②連結可能匿名化する
5. 同意を得ないで，連結可能匿名化する（4=②）場合	(1)資料提供時の同意が遺伝子解析研究の目的と相当の関連性を有すると<br>□合理的に認められる<br>（研究の目的を提供者へ通知または公表するための措置を講じること）<br>□認められない（(2) ー (5) への記載が必要）<br>(2) 解析により提供者等に危険や不利益が及ぶ可能性が<br>□ ①極めて小さい（理由： ）<br>□ ②ある<br>(3) 研究が公衆衛生の向上のために<br>□ ①必要とされる（理由： ）<br>□ ②必要とされない<br>(4) 他の方法では事実上研究の実施が<br>□ ①不可能（理由： ）<br>□ ②可能<br>(5) 検体提供者または代諾者等の同意を得ることが<br>□ ①困難である（その理由： ）<br>②容易である
6. 既提供検体の取得時期	□ ①平成13（2001）年3月31日以前<br>□ ②平成13（2001）年4月1日以降

VII. 検体・遺伝情報の共同研究機関以外の公的機関への提供（細胞・遺伝子・組織バンクへの

遺伝子解析計画名：運動と補助食品摂取の併用による生活習慣病予防効果の検証ー血液細胞のＤＮＡメチル化を調べそのエピジェネティックスの側面からの検証

9

寄託の場合，本欄への記載は不要。IX-3 に記載のこと）

1. 検体またはそれから得られた遺伝情報を他の公的研究機関に提供するか？	□ ①提供する（2 以下への記載が必要）<br>（提供が必要な理由：　　　　　　）<br>■②提供しない
2. 提供する（1=①）場合，提供先機関名	
3. 提供する（1=①）場合，提供元において匿名化するか？	□ ①連結不可能匿名化する<br>□ ②連結可能匿名化する<br>□ ③匿名化しない（理由：　　　　　　）
4. 検体等を提供する（1=①）場合，提供先機関において遺伝子解析を行うか	□ ①行う<br>□ ②行わない
5. 検体等を提供する（1=①）場合，反復，継続して提供をおこなうか？	□ ①行う<br>□ ②行わない

VIII. 営利団体等への提供（解析を外部委託する場合等を含む）

1. 検体またはそれから得られた遺伝情報を他の営利団体，民間の機関に提供するか？	□ ①提供する（2 以下への記載が必要）<br>（提供が必要な理由；　　　　　　）<br>■②提供しない
2. 提供先機関名	
3. 提供元における匿名化の方法	□ ①連結不可能匿名化<br>□ ②連結可能匿名化
4. 提供先における責任者の氏名，責任体制，予定する契約の内容	

IX. 検体等の保存

1. 実施期間中の検体の保存	(1)研究期間中の試料等の保存の有無	■ ① 機関内で保存する（→以下への記載が必要）<br>□ ② 共同研究機関で保存する（→以下への記載が必要）<br>□ ③ 保存しない　（→4 への記載が必要）
	(2)保存する場合（(1)＝①または②），その方法と保存期間	□ ① 血液・組織等のまま保存（保存期間　　年　　月　　日まで）<br>■ ② 核酸を抽出して保存（保存期間　　年　　月　　日まで）<br>□ ③ 細胞を不死化して保存（保存期間　　年　　月　　日まで）<br>□ ④ その他（　　　　　　）
	(3)保存する場合（(1)＝①または②），その必要性	■ ① 遺伝子解析結果の正確性を期するため，再検に備える目的で<br>□ ② その他（　　　　　　）
	(4)試料等の保存の管理責任者	□ ① 本学の研究実施責任者<br>■ ② ①以外の研究者が管理する（→管理責任者氏名を記載）<br>所属：加齢生物学分野<br>職名：助教<br>氏名：澤下仁子
2. 実施期間後の検体の保存	(1)研究終了後の試料等の保存の有無	■ ① 機関内で保存する（場所：医学部内 2011 年から 2016 年）<br>□ ② 共同研究機関で保存する<br>□ ③ 保存しない

10

	(2)保存する場合（(1)＝①または②），その方法と保存期間	□ ① 血液・組織等のまま保存<br>■ ② 核酸を抽出して保存<br>□ ③ 細胞を不死化して保存<br>□ ④ その他（ ）
	(3)保存する場合（(1) ＝①または②），その必要性・理由	■ ① 将来の研究ための貴重な試料・資料とするため（予測される研究内容：今回と同様 ）<br>□ ② その他（ ）
	(4)保存の場合（(1) ＝①または②），匿名化の種類	□ ① 連結不可能匿名化<br>■ ② 連結可能匿名化
3. 細胞・遺伝子・組織バンクへの検体等の寄託	(1) 寄託の予定	□ ①予定している（(2) (3) への記載が必要）<br>■ ②予定していない
	(2) 寄託を予定している（(1) =①）具体的な寄託先	□ ①決まっている（バンク運営機関名・責任者氏名： ）<br>□ ②決まっていない（決定した際，報告する）
	(3) 寄託を予定している（(1) =①）場合，匿名化の方法	□ ①連結不可能匿名化
4. 検体等を廃棄する際の方法		□ ① 匿名化のまま，密封容器に廃棄<br>■ ② 匿名化のまま，焼却処分<br>□ ③ 匿名化のまま，紙データについてはシュレッダー等を用いて廃棄し，電子データについてはデータを完全に消去する<br>□ ④ その他（ ）

X. 遺伝カウンセリング

1. 遺伝カウンセリングの必要性	□ ①原則として必要<br>■②場合により必要<br>□ ③必要ない（理由： ）
2. 必要な（1=①か②）場合，遺伝カウンセリングの担当施設，担当者名（資格，教育研修歴等）	■①信州大学医学部附属病院遺伝子診療部，福嶋義光（臨床遺伝専門医）他<br>□ ②その他（ ）

XI. 研究に係る資金源

研究に係る資金源	■ ① 運営費交付金（教育研究費）<br>■ ② 省庁等の公的研究費（具体的に：文部科学省　平成２３年度特別経費）<br>□ ③ 共同研究経費・受託研究経費<br>□ ④ 委任経理金（奨学寄附金）<br>□ ⑤ 研究費はとくに必要としない<br>□ ⑥ その他（具体的に： ）

XII. 本研究と企業・団体との関わり

本研究と企業・団体との関わり	■ ① 本研究に企業等は関与しない

遺伝子解析計画名：運動と補助食品摂取の併用による生活習慣病予防効果の検証ー血液細胞のＤＮＡメチル化を調べそのエピジェネティックスの側面からの検証

	□ ② 共同研究として実施（相手先：　　　　　　　）<br>［共同研究契約書の写しを添付のこと］<br>□ ③ 受託研究として実施（相手先：　　　　　　　）<br>［受託研究契約書の写しを添付のこと］<br>□ ④ 本研究に関与する企業等はあるが，共同研究・受託研究として実施しない（相手先：<br>関与の具体的内容：　　　　　　　）<br>（注：企業等へ研究成果などを提供する場合，個人情報保護・知的財産保護のため，共同研究・受託研究への移行が必要となる．<br>また，研究の性質によっては，補償等の関係から共同研究・受託研究への移行が必要となる．）

XIII. 起こりうる利害の衝突

28. 起こりうる利害の衝突[i]<br>（該当するものすべてにチェック）<br><br>注：本項目は，情報開示による利益相反への適切な対応を目的としており，該当した場合でも本学が許容できる範囲内であれば，特に研究計画の変更等は必要有りません．（研究推進・研究者保護が趣旨）．<br>本申請書末尾の解説を参照の上，記載すること．<br>なお，本項目に関する事項は必要に応じて「国立大学法人信州大学臨床研究に係る利益相反マネジメント規程」に従って審議されます．	□ ① 研究実施責任者及び研究者等が，本研究に関与する企業等から，本研究への関連の有無に関わらず共同研究費，受託研究費，委任経理金（奨学寄附金）等の研究資金を受け入れている．<br>□ ②本研究に関与する企業等との間に，本研究への関連の有無に関わらず機器や消耗品等の提供，依頼試験・分析，研究員の受入等がある．<br>（具体的に：　　　　　　　）<br>□ ③ 研究実施責任者及び研究者等が，本研究に関与する企業等との間に，役員・顧問等の非常勤を含む雇用関係，研究資金以外の謝金・報酬・給与の支払い等の経済的利益，知的財産権（特許や実用新案等）の共有・実施許諾・譲渡がある．または，当該企業の株式（未公開株・ストックオプションを含む）を所有している．<br>（個人情報を保護できる範囲で具体的に：　　　　　　　）<br>□ ④ 研究実施責任者及び研究者等の，2親等以内の親族が，本研究に関与する企業等との間に，研究費の受入，報酬・給与等の経済的利益，経営関与，知的財産権（特許や実用新案等）の共有・実施許諾・譲渡がある．または，当該企業の株式（未公開株・ストックオプションを含む）を所有している．<br>（個人情報を保護できる範囲で具体的に：　　　　　　　）<br>□ ⑤ 本研究の実施によって，本務である教育・研究・診療等に支障をきたす可能性がある．<br>（具体的に：　　　　　　　）<br>■ ⑥ 利害の衝突は起こり得ない

XIV. 特記事項

1. 特記事項の有無	□ ①治療法・予防法のない疾患（解析が必要な理由：　　　　　　　）<br>■②審査に迅速性が要求されるケース（理由：9月上旬開始予定）<br>□ ③個々の症例に対する申請　前例（□有，□無）<br>□ ④共同研究であって，既に主たる研究機関において倫理審査委員会の承認を受けた研究計画を分担研究機関として実施しようとするもの（承認証明書の写しを添付する）

12

	□ ⑤既に承認されている遺伝子解析計画に準じて類型化されている計画の申請（承認された遺伝子解析計画名・承認番号：　　　　　）<br>□ ⑥上記の①～⑤に該当せず

XV．実施計画書の開示

1．本遺伝子解析実施計画書の開示の可否	■①可<br>□ ②一部不可<br>□ ③不可
2．一部不可（1=②）の場合，開示を望まない項目番号	
3．（一部）不可（1=②か③）の時，開示を望まない理由	□ ①検体提供者の人権に支障が生じる可能性がある<br>□ ②研究の独創性に支障が生じる可能性がある<br>□ ③知的財産権の保護に支障が生じる可能性がある<br>□ ④その他（詳しく説明：　　　　　　　　　　）

XVI．その他

自由記載欄（審査に対する希望など）	

---

[i]「利害の衝突」とは，研究者等が研究の実施や報告の際に，金銭的な利益やそれ以外の個人的な利益のためにその専門的な判断を曲げてしまう（もしくは曲げたと判断される）ような状況や，当該研究の実施によって本務である教育・研究・診療等に支障をきたすような状況を示す．（前者を狭義の「利益相反」，後者を「責務相反」という．）

この狭義の「利益相反」は，研究者やその親族等が資金提供や研究依頼のあった者・団体（政府，財団，企業等）から臨床研究に係る資金源の他に機器や消耗品等の提供を受けること，実施料を受け取ること，その株式を所有（未公開株やストックオプションを含む）すること，特許権を共有・譲渡されること，講演料や著述料の支払いを受けていること等である．

それ以外の利害の衝突とは，研究者やその親族等が資金提供や研究依頼のあった者・団体との間に顧問等の非常勤を含む雇用関係があることや，親族や師弟関係等の個人的関係があることなど，研究者等の関連組織との関わりについての問題などが考えられる．

上記の様な利害の衝突の中でも，明示的に確認することができる①資金源等の金銭上の利害の衝突②研究者等の関連組織との関わりについては少なくとも記載するべきである．
